# Evidence for the Vacated Niche Hypothesis in Parasites of Invasive Mammals

**DOI:** 10.1002/ece3.70959

**Published:** 2025-02-10

**Authors:** Annakate M. Schatz, Andrew W. Park

**Affiliations:** ^1^ Odum School of Ecology University of Georgia Athens Georgia USA; ^2^ Center for the Ecology of Infectious Diseases University of Georgia Athens Georgia USA; ^3^ Department of Infectious Diseases, College of Veterinary Medicine University of Georgia Athens Georgia USA

**Keywords:** enemy release, host–parasite, invasive species, macroecology, null model, vacated niche

## Abstract

Species redistribution and invasion are becoming increasingly common due to climate change and anthropogenic impacts. Understanding the resultant shifts in host–parasite associations is important for anticipating disruptions to host communities, disease cycles, and conservation efforts. In this paper, we bring together the enemy release and vacated niche hypotheses to relate parasite acquisition and retention, two distinct yet intertwined processes that play out during host invasion. Using the Global Mammal Parasite Database, we test for net enemy release based on differences in parasite species richness, and we develop a novel taxonomic null modeling approach to demonstrate that parasites fill vacated niches. We find evidence of net enemy release, and our taxonomic null models indicate replacement of lost parasites by taxonomically similar acquired ones, over and above what might be expected by chance. Our work suggests that both enemy release and vacated niche hypotheses provide valuable frameworks through which to understand and predict changing host–parasite associations, which may include insights on how climate change and anthropogenic influences perturb and reorganize communities and ecosystems.

## Graphical abstract

With species redistribution and invasion becoming more common, it is important to understand the resultant shifts in host–parasite associations. In this investigation of invasive terrestrial mammals, we develop a novel taxonomic null modeling approach demonstrating replacement of lost parasites by taxonomically similar acquired ones, over and above what might be expected by chance. Our work suggests that the vacated niche hypothesis provides a valuable framework through which to understand and predict changing host–parasite associations.

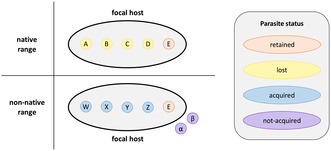

## 1 Introduction

**FIGURE 1:**
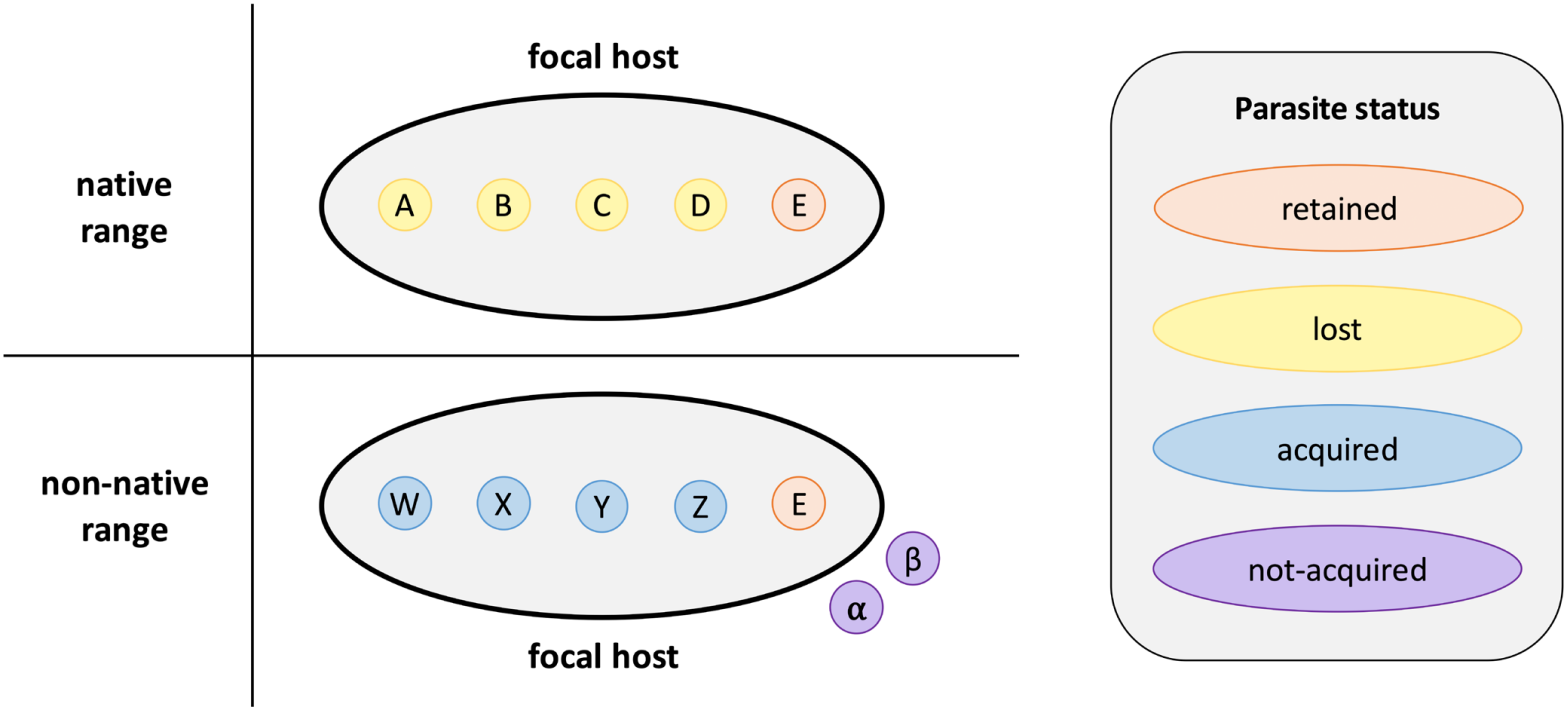
Conceptual diagram of parasite fates with host invasion: Retention, loss, acquisition, and non‐acquisition. The top row shows parasites associated with the focal host (depicted by the shaded oval) in its native range. The bottom panel shows parasites associated with the host in its non‐native range, along with those not‐acquired parasites that do not associate with the focal host.

Species redistribution and invasion are becoming increasingly common due to climate change, anthropogenic influences such as urbanization or deforestation, and accidental or intentional translocations, among other factors (Chen et al. 2011; Hellmann et al. 2008; Levine and D'Antonio 2003; Mainka and Howard 2010). When species are introduced to new communities, they might bring parasites with them from their native ranges or form new parasite associations in the non‐native ranges (Kelly et al. 2009; Lymbery et al. 2014; Morales‐Castilla et al. 2021; Schatz and Park 2021, 2023; Strauss et al. 2012). These dynamics can be divided into four possible parasite fates: retention (carried with the host during invasion), loss (not carried with the host during invasion), acquisition (newly picked up by the host post‐invasion), and non‐acquisition (not picked up by the host post‐invasion; Figure 1; Schatz and Park 2021; Schatz and Park 2023). For example, the invasive *Python bivattus* (Burmese python) in southern Florida retained a pentasomid parasite (*Raillietiella orientalis*) during invasion, which has now spread hundreds of kilometers via spillover to competent native hosts (Miller et al. 2020). In the Pacific Islands, on the other hand, an invasive gecko *Hemidactylus frenatus* acquired several new parasites from the native house gecko *Lepidodactylus lugubris* , which likely played a role in the former's displacement of the latter (Kelly et al. 2009). It is important to understand the dynamics of changing host–parasite associations for a number of reasons. Hosts carrying pathogens from their native ranges or acquiring established diseases in the non‐native ranges can disrupt disease cycles and alter disease impacts through spillover or spillback, respectively (Kelly et al. 2009; Lymbery et al. 2014). Sometimes, these dynamics will lead to conservation and public health issues with parasites as direct or indirect culprits (Iglesias et al. 2015; Lloyd‐Smith et al. 2009).

One key theoretical framework through which scientists have studied host–parasite community perturbations and the underlying processes is the *enemy release hypothesis*, which states that invasive species are successful in part because they escape natural enemies from their native range, such as predators or parasites (Mitchell and Power 2003; Torchin et al. 2001, 2003). As a complementary theory, researchers have also put forward the novel weapons hypothesis to describe the impacts of parasite coinvasion with their hosts; while this has largely been developed for plants, it can apply broadly across any invasive host (Callaway and Ridenour 2004; Vilcinskas 2015). Research in these areas has contributed greatly to our understanding of host–parasite dynamics during invasions, for example, by accounting for both parasite loss and acquisition to capture *net enemy release* (Torchin et al. 2003; Marzal et al. 2011). Considering both loss and acquisition of parasites also raises the possibility of *transient enemy release*—that is, temporary invasion success facilitated by parasite loss which is subsequently overwhelmed by parasite acquisition (Flory and Clay 2013; Gendron et al. 2011; Kołodziej‐Sobocińska et al. 2018). In this paper, we supplement such quantitative analyses of host–parasite invasion dynamics with qualitative considerations based on the vacated niche hypothesis, described below.

Our hominid ancestors offer an early indication of the importance of open niches for parasites. As hominids transitioned from arboreal to land‐based living, they encountered new parasites, including schistosomes (Despres et al. 1992). Molecular evidence suggests that in what is now Africa, hominids first acquired *Schistosoma mansoni* from other animals; as hominid expansion continued into what is now Asia, this parasite was lost, but the related *S. japonica* was acquired from different animal species (Despres et al. 1992). Clearly, an unexploited niche existed in early hominids and related parasite species were able to use it. Far from being confined to evolutionary history, though, the *vacated niche hypothesis* appears to be at work today (Lloyd‐Smith 2013). Connected to enemy release, this hypothesis posits that the extinction, elimination, or loss of one parasite species leaves an ecological niche open that another species might invade and exploit, most likely a related or otherwise similar species (Lloyd‐Smith 2013). The hypothesis draws on the observations that related parasite species compete more strongly within hosts, including direct competition for space and resources and indirect (immune‐mediated) apparent competition (Bashey 2015; Pedersen and Fenton 2007; Mideo 2009); the niche overlap of related parasites suggests that host susceptibility, resistance, or tolerance to one parasite is likely similar for related parasites, and related parasites may exploit such niches more successfully in the absence of competition. The profile of parasite niches has been developed in a number of systems (Cizauskas et al. 2017; Cumming 1999; Lira‐Noriega and Peterson 2014; Maher et al. 2010) and is potentially more complex than for free‐living species because it includes host and environmental dimensions. The vacated niche hypothesis has largely been developed around a few specific case studies (mostly viruses), rather than as a general phenomenon. For example, the eradication of smallpox has led to new concerns about zoonotic spillover of monkeypox into that niche, with recent monkeypox cases in humans seeming to support such worries (Lloyd‐Smith 2013; Reynolds et al. 2012; Weyer and Blumberg 2022). Cases of peste des petits ruminants (PPR) have caused significant economic disruption in parts of Africa and Asia; recent increases in disease circulation might be related to the 2011 eradication of rinderpest, another closely related morbillivirus (Baron et al. 2011; Lembo et al. 2013; Roeder et al. 2013).

Despite such useful work, we are left with the open question of whether these case studies are idiosyncratic or represent the tip of the iceberg. Ample evidence exists for vacated niches among parasites, with the process of parasite loss underlying enemy release as a prime example (e.g., Johnson et al. 2020; Marr et al. 2008), but it is not yet clear if parasite dynamics consistently follow vacated niche predictions. There are numerous examples of competitive exclusion occurring between parasite species seeking to exploit an animal host population, including humans (Molyneux et al. 2014), indicating the potential importance of the open niche. However, parasites seeking to colonize vacated niches still face numerous obstacles, such as host exposure opportunity, host susceptibility (especially if the host maintains a level of cross‐immunity due to related infections), and transmission barriers (such as host population size in the case of species invasions; Johnson et al. 2015). In this paper, we aim to establish general vacated niche patterns by moving beyond the well‐cited case studies of viruses discussed above (smallpox/monkeypox and rinderpest/PPR). We include five parasite types, as well as considering other parasite traits (taxonomy, host specificity, and transmission modes).

With the theories outlined above in mind, we come to the crux of this work: against the backdrop of enemy release, the dynamics of parasite loss and acquisition predicted by the vacated niche hypothesis have the potential to shape and inform the trajectory of host–parasite associations during invasion. Enemy release (through parasite loss) creates vacated niches for other parasites (as seen in Johnson et al. (2020); Marr et al. (2008), for example). Filling vacated niches (through parasite acquisition) can then lead to transient enemy release. Thus, the vacated niche hypothesis allows us to relate parasite acquisition and retention, two distinct yet intertwined processes that are often confounded in enemy release work. Note that we are thinking about these processes at the level of the host population and parasite community, rather than expecting one‐for‐one parasite swaps in a single host individual to match the vacated niche hypothesis. This is part of the value of taking a macroecological approach, as we do here, to combine data across hosts, time, and sampling events (Stephens et al. 2016). In this paper, we use data from the Global Mammal Parasite Database on five invasive mammals (red deer, common raccoon, chamois, wild boar, and red fox) whose invasions were facilitated by human translocation of animals (Ikeda et al. 2004; Long 2003; Martínková et al. 2012). We aim to evaluate support for the vacated niche hypothesis across five taxonomic groups of parasites (arthropods, bacteria, helminths, protozoa, and viruses) and multiple parasite traits, while also assessing evidence for net enemy release in invasions across the globe. As part of our vacated niche investigation, we developed a novel taxonomic null modeling approach that leverages information on parasite relatedness and has the potential to be applied to other data in the future.

## 2 Methods

### 2.1 Data Preparation

This work used host–parasite association data from the Global Mammal Parasite Database (GMPD), a comprehensive collection of host–parasite sampling records gathered from the scientific literature (Stephens et al. 2017). We identified terrestrial mammal hosts sampled in both native and non‐native ranges according to the GMPD; these are referred to as our focal hosts. GMPD records are geogreferenced but do not provide spatially explicit host ranges. Thus, we translated the GMPD sampling points into range areas using buffered IUCN range data (IUCN 2016). Non‐native ranges were defined as those ecoregions containing GMPD sampling records outside the native range and were further refined using zoogeographic realms (Holt 2013), so that invasions in disparate parts of the globe were not analyzed as a single event. Figure S1 shows native and non‐native range maps for each focal host included in these analyses.

These ranges allowed us to define native and non‐native host–parasite communities for each focal host by collecting all GMPD records located within the native and non‐native ranges. We then categorized the specific fate of each parasite upon host invasion (Figure 1). *Retained* parasites are those sampled from the focal host in both its native and non‐native ranges; *lost* parasites are those sampled from the focal host only in its native range (Schatz and Park 2023). *Acquired* parasites are those sampled from the focal host only in its non‐native range; *not‐acquired* parasites are those present in the non‐native range but not sampled from the focal host (Schatz and Park 2021).

For the purposes of this study, we consider five focal hosts (two carnivores and three ungulates; Table 1). These hosts were identified in previous work as those with sufficient GMPD data available to analyze parasite acquisition and retention; hosts had to have at least 5 acquired or retained parasites to be included in the respective study, as well as meeting standards for samples collected in the native and non‐native ranges (details in Schatz and Park (2021); Schatz and Park (2023)). For the current study, only hosts with both acquisition and retention data are considered, which further limited host inclusion. The five hosts meeting our data standards are associated with 398 unique parasites (retained, lost, and acquired). We note that for these hosts, the level of study of their lost parasites (both number of GMPD‐recorded publications per parasite and number of host animals per study) as they move to their invasive ranges is similar to that for both retained parasites and parasites not acquired from the invasive range parasite pool (Table S1). In contrast, parasites acquired in the invasive range are typically less studied for the focal hosts (Table S1), which implies that future studies of these hosts in their invasive range may uncover further host–parasite associations. Importantly, the lost and not‐acquired statuses are inferred from the absence of sampling records, and these categories are not conspicuously underpublished or undersampled (Table S1). See Tables S1 and S2 for a summary of sampling effort and taxonomic distribution of GMPD data underlying our definitions of parasite fates.

**TABLE 1:** Focal hosts' names, native and non‐native ranges by continent, and parasite species richness (PSR) in native and non‐native ranges, further subdivided into counts of retained, lost, and acquired parasites.

Focal host	Native range	Non‐native range	Native PSR	Non‐native PSR	Retained	Lost	Acquired
*Cervus elaphus* (red deer)	Europe	North America	104	31	17	87	14
*Procyon lotor* (common raccoon)	North America	Asia	126	18	7	119	11
*Rupicapra rupicapra* (chamois)	Europe, Asia	Europe	58	22	13	45	9
*Sus scrofa* (wild boar)	Europe, Asia, Africa	North America	76	16	9	67	7
*Vulpes vulpes* (red fox)	Europe, Asia, Africa	North America	127	38	25	102	13

All data preparation, analysis, and modeling were done in R v3.5.1–4.2.1 (R Development Core Team 2008). For further methodological detail on data preparation, see Schatz and Park (2021, 2023). Data and reproducible code for all methods are available on Dryad (doi: 10.5061/dryad.37pvmcvsp) and Zenodo (doi: 10.5281/zenodo.14756595).

### 2.2 Enemy Release

To assess support for the enemy release hypothesis, we first analyzed the change in parasite species richness (PSR) for each focal host from its native to non‐native range (Table 1). We used a phylogenetic paired *t*‐test (phyl.pairedttest function from the phytools package v1.0‐3; Revell 2012) using the “best dates” version of the mammalian supertree from Bininda‐Emonds (2007).

We then explored potential drivers of net enemy release using simple linear regressions. We built four response variables to capture different aspects of the change in PSR. First we used a simple proportional change in PSR; this was calculated as 1—non‐native PSR/native PSR. We also calculated beta diversity, used to measure changes in parasite community composition for each focal host. Following Herrera et al. (2019), we compartmentalized beta diversity values into turnover (contribution to beta diversity of new parasite associations formed during invasion) and nestedness (contribution to beta diversity of unchanged parasite associations). These were calculated with the beta. pair function in the betapart package (v1.5.6), using “family” argument “sorensen” (Baselga 2023). Turnover and nestedness were analyzed as proportions of full beta diversity to ensure comparability of values across focal hosts.

We also collected four predictor variables: time since invasion (estimated from the literature; Ikeda et al. 2004; Long 2003), population density (Jones et al. 2009), home range area (log‐transformed for modeling; Jones et al. 2009), and environmental dissimilarity between ranges. Predictors were used one at a time for each response variable in turn. We expect that time since invasion might affect how many parasites are retained or acquired, as hosts settle in to their invasive range (as discussed in, for example, Gendron et al. 2011; Kołodziej‐Sobocińska et al. 2018). Population density and home range area values are included as species‐level traits that characterize how each host species tends to occur in space, using PanTHERIA data that generalizes across populations (Jones et al. 2009). Finally, environmental dissimilarity captures potential abiotic challenges to or facilitation of parasite establishment and circulation. This predictor was defined as the mean Bray–Curtis distance between native and non‐native ranges, calculated using the vegdist function in the vegan package (Oksanen 2022); ranges were described using 19 bioclimatic variables available from WorldClim for 1970–2000 (Hijmans et al. 2005).

### 2.3 Vacated Niche

We evaluated evidence for the vacated niche hypothesis based on multiple parasite traits: parasite taxonomy, phylogenetic specificity, parasite type, and transmission mode.

#### 2.3.1 Parasite Taxonomy

**FIGURE 2:**
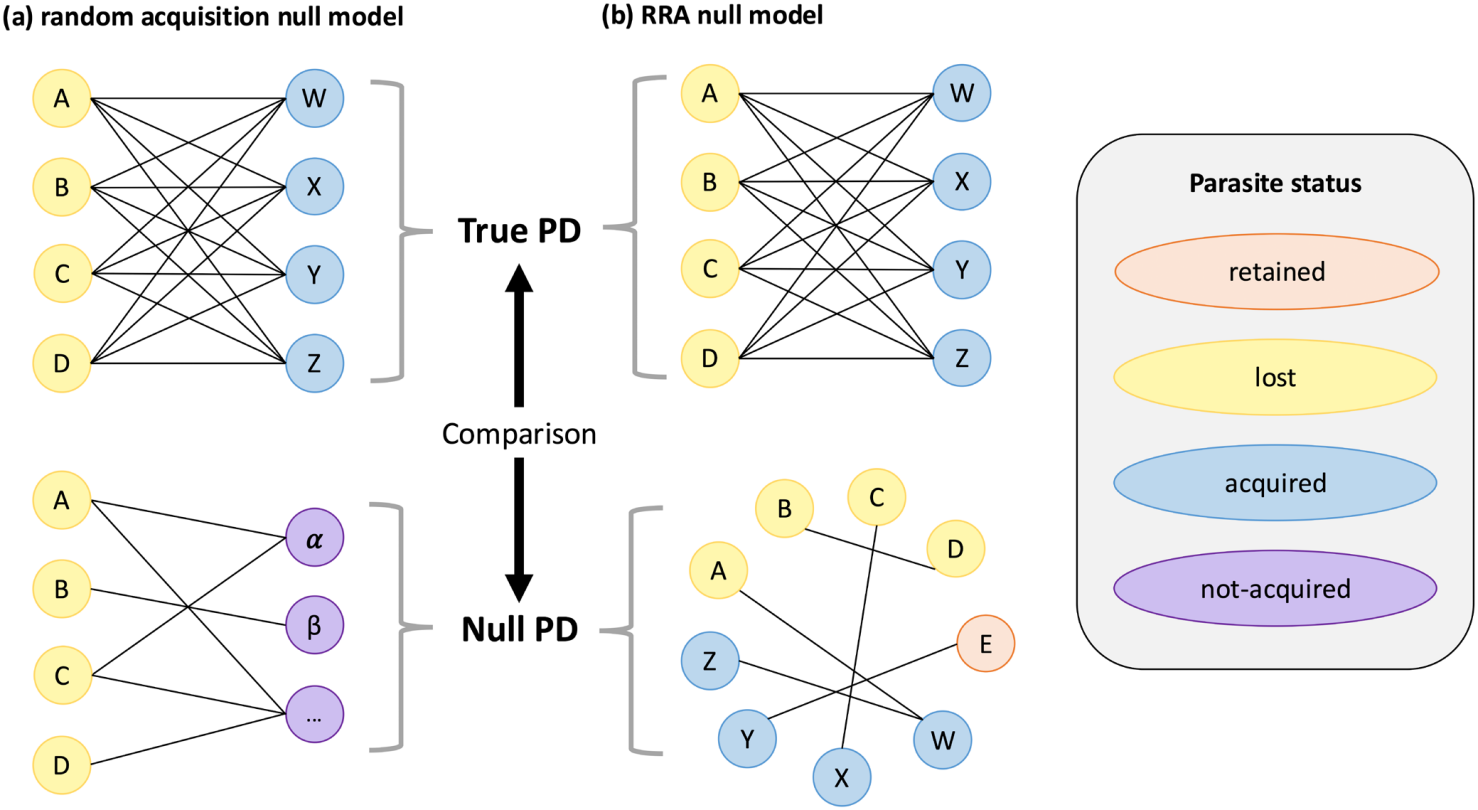
Conceptual diagrams of null models. (a) Random acquisition null model: Are lost parasites more related to acquired parasites than not‐acquired parasites? (b) Random‐relative‐to‐associations (RRA) null model: Are lost and acquired parasites more related than other parasite pairs that infect the host? In both panels, thin black lines indicate calculations of pairwise distance between parasites; a random representative subset of the possible calculations is shown for the null PDs, as sampling is not exhaustive. PD = pairwise distance based on taxonomic trees of parasites. See Methods and Figure 1 for parasite status definitions.

Most substantially, we investigated parasite taxonomy within each of the five parasite types included in our analyses (arthropods, bacteria, helminths, protozoa, and viruses). We developed two null models to test for patterns in the taxonomy of lost and acquired parasites, described in detail below (Figure 2). The first of these null models we refer to as the *random acquisition null model*; it seeks to answer the question: are lost parasites more related to acquired parasites than to not‐acquired parasites? The second null model is the *random‐relative‐to‐associations (RRA) null model*, which addresses the question: are lost and acquired parasites more related to each other than random parasite pairs that infect the host? These two null models complement each other by considering parasite acquisition in relation to both the entire pool of potentially acquired parasites (i.e., the component parasite community of the invaded range) and those particular parasites known to form associations with the focal host.

#### 2.3.2 Taxonomic Data

Taxonomic data (kingdom, phylum, class, order, family, genus, and species) for individual parasites was queried in R from the NCBI and ITIS databases (taxonomy function from the myTAI package v0.9.3; Drost et al. 2018). Where parasite data was unavailable via the aforementioned queries, 49 entries were completed manually via web search (citations in code). Next we calculated the true minimum pairwise distances (PDs) between all pairs of acquired and lost parasites within each parasite type. The use of this metric is predicated on the idea that, while several parasite species may be lost and acquired by a host species, vacated niches are exploited by related parasites, which are anticipated to be functionally and immunogenically similar (Bashey 2015; Pedersen and Fenton 2007). The detection of such a trend may be achieved by measuring closest relatives between pairs of lost and acquired parasites. The distances refer to the number of taxonomic levels separating pairs of parasite species, established using unit branch lengths between levels. Similar methods have been used to approximate phylogenetic relationships between host species, especially in cases with incomplete phylogenetic information (Dáttilo et al. 2020; Morand and Poulin 2003; Poulin 2005), as is the case for the parasite taxa in our study.

#### 2.3.3 Taxonomic Null Models

We then set up each of our null models, which drew random parasite pairs with replacement and calculated minimum null PDs. The *random acquisition null model* used 1000 draws from the focal host's pool of lost and not‐acquired parasites (potential non‐native associations), while the *RRA null model* used 100 draws from the focal host's collection of retained, lost, and acquired parasites (known associations). Finally, we calculated *z*‐scores for each null model by comparing the true PDs to the null distributions. Negative *z*‐scores mean the lost and acquired parasites are more related than expected under the null models (support for the vacated niche hypothesis); positive *z*‐scores mean they are less related than the null expectation. These scores have the additional benefit of being expressed in units of normalized standard deviations, aiding both comparison with potential future studies and appraising important variations from expectations. In particular, *z* = −1.645 approximates the threshold above which 95% of observations lie, under a “one‐tailed” expectation that lost and acquired parasites are more related to each other.

We note that for the random acquisition null model, we included all not‐acquired parasites as potential associations for a given focal host, regardless of their particular host(s) in the invaded community. While it is true that the invasive focal host might be more likely to fill a vacated niche by acquiring parasites from more closely related hosts (Schatz and Park 2021), excluding distantly related hosts from contributing to the pool of not‐acquired parasites may be counterproductive. Many parasites exhibit a large phylogenetic span, within which they are aggregated in infecting closely related hosts; in other words, parasites might infect hosts both closely and distantly related to our focal host and thus would be included even if distantly related hosts were dropped (Park et al. 2018). There is also the fact that not all host–parasite associations are known (Dallas et al. 2017a), so a parasite that does infect a closely related host might be incorrectly excluded from consideration, simply because that association has not yet been detected. Lastly, competitive exclusion principles (Martin and Ghalambor 2023) may mean it is unlikely for closely related host species to co‐occur at appreciable population sizes, invalidating the premise of parasite transmission from related hosts in some cases.

#### 2.3.4 Other Parasite Traits

We considered the phylogenetic specificity of lost and acquired parasites, using phylogenetic z‐scores from Park et al. (2018). These data, which account for divergence times between host species, were analyzed with the Mann–Whitney *U* test to compare the two distributions of specificities.

In addition, we evaluated two categorical variables from the GMPD: parasite type (arthropod, bacteria, helminth, protozoa, and virus) and transmission mode (non‐exclusive categories of close contact, non‐close contact, intermediate, and vector; Stephens et al. 2017). Data for these traits were analyzed with the Fisher test to accommodate low sample sizes, comparing the proportions of a focal host's lost and acquired parasites classified in each category.

## 3 Results

Lending support to the enemy release hypothesis, we found a significant decrease in PSR across our five focal hosts from the phylogenetic paired *t*‐test (*t* = 6.11, degrees of freedom = 2, *p* = 0.026; Table 1, native and non‐native PSR columns). We did not, however, find significant results in our analysis of potential drivers of net enemy release patterns (time since invasion, population density, range area, and environmental dissimilarity) for any combination of predictor and response (Table S3). In all cases, coefficients were close to zero and no *p*‐value was less than 0.1 (Table S3). We speculate that some of these negative results might be due to our low sample size (five focal hosts).

**FIGURE 3:**
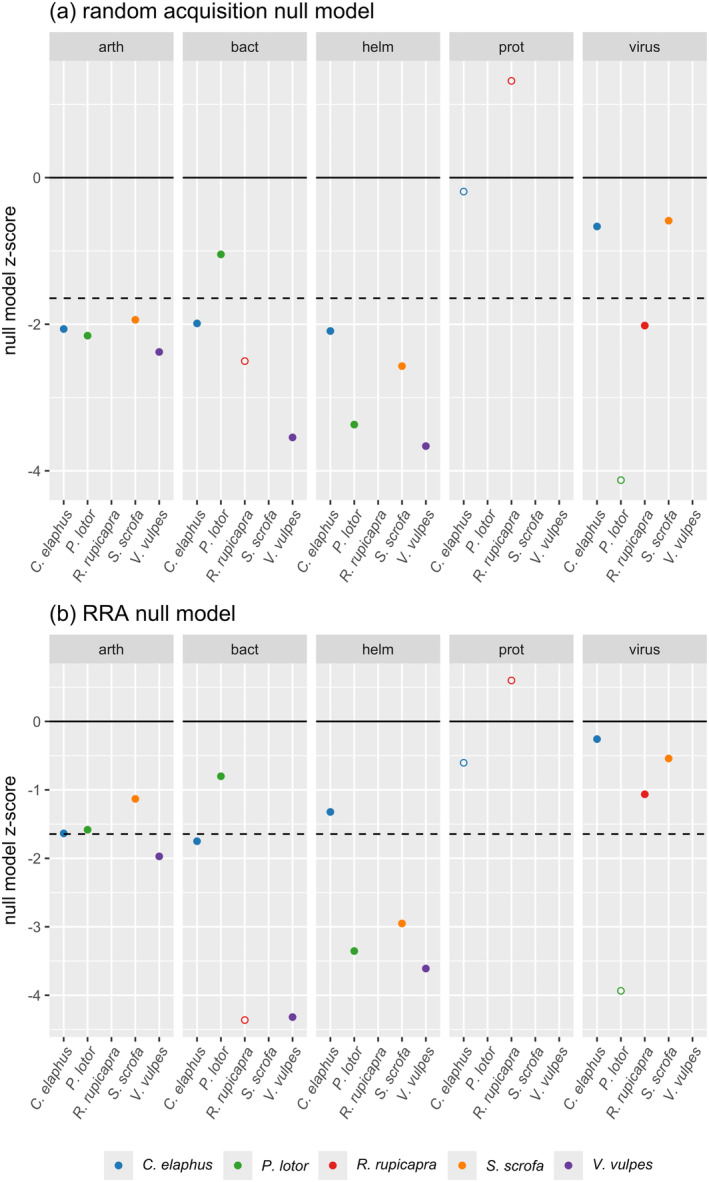
Results from (a) random acquisition null models and (b) random‐relative‐to‐associations (RRA) null models, using minimum pairwise distance (PD) and faceted by parasite type. See Methods and Figure 2 for null model descriptions. Open points denote null model *z*‐scores based on fewer than 10 pairwise distances; closed circles denote null model *z*‐scores based on 10 or more pairwise distances. Missing points indicate no loss and/or acquisition of a parasite type by the focal host species, meaning null model *z*‐scores could not be calculated. The dashed line marks a *z*‐score of −1.645, the threshold for significance under a “one‐tailed” expectation that lost and acquired parasites are more related to each other.

Overall, our results strongly support the vacated niche hypothesis. The random acquisition null model returns negative *z*‐scores for four of the five parasite types (all except protozoa); this indicates replacement of lost parasites by taxonomically similar acquired ones, over and above what might be expected by chance (Figure 3a). Similarly, the RRA null model has negative *z*‐scores for all five parasite types, suggesting greater taxonomic similarity between a focal host's lost and acquired parasites than randomly selected host‐associated parasites (Figure 3b). Across both null models, the majority of null model *z*‐scores are not just negative, but significantly so at *z* < −1.645; the median quantile of these *z*‐scores is 0.012 for the random acquisition null model and 0.015 for the RRA null model (Table A4; Kembel et al. 2010). It is interesting to observe the different levels of null model *z*‐score significance across parasite types—for example, helminths (and to a lesser extent arthropods) generally have lower *z*‐scores than viruses (Figure 3). We also note that some focal hosts and parasite types have low sample sizes for lost and/or acquired parasites, which reduced the number of comparisons used to calculate null model *z*‐scores (see open points in Figure 3). It is possible that parasite groups with higher richness and thus more comparisons are more likely to show significant *z*‐scores, but a Spearman's correlation test found no significant correlation between *z*‐score and number of comparisons (*rho* = −0.309, *p* = 0.213); the small negative values of *rho* do support our initial logic though, so this is a sample size artifact of which researchers should be aware.

**FIGURE 4:**
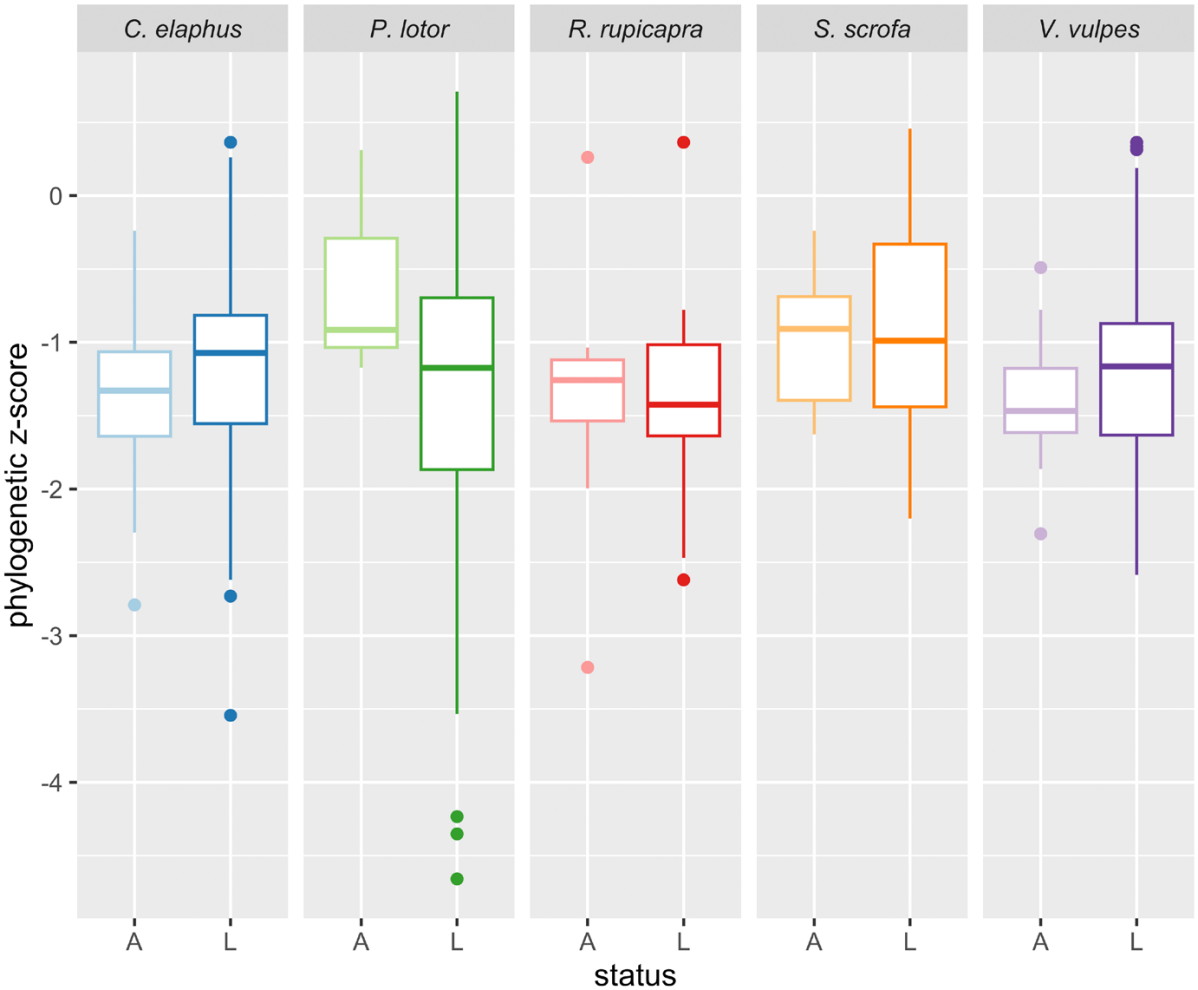
Boxplot of phylogenetic host specificities for acquired (A) and lost (L) parasites, grouped by focal host. Specificity is represented by a phylogenetic *z*‐score (see Methods for details).

We found no significant differences in the phylogenetic specificity distributions of lost and acquired parasites; we note that *Procyon lotor* comes closest (*p* = 0.065; Figure 4 and Figure S2). For parasite type and transmission mode, only *Rupicapra rupicapra* 's lost and acquired parasites differ (parasite type: *p* < 0.001; transmission mode: *p* = 0.016; Figures S3 and S4).

## 4 Discussion

Overall, we found support for the key requisite of the enemy release hypothesis, namely, a reduced number of parasite species in the invaded versus native range of invasive mammals. This is true even with explicit consideration of parasite acquisition post‐invasion, with the net effect representing a possible benefit to the invasive species, thanks to lower levels of PSR. It is worth noting, however, that actual enemy release experienced by these invaders could be less than that implied by the stark differences in PSR. Acquisition numbers are quite low, which might suggest undersampling or insufficient time for parasites to adapt to and colonize invasive hosts (Ikeda et al. 2004; Long 2003). Furthermore, PSR is not necessarily a full picture of parasite pressure and might not adequately capture processes that actually lead to host demographic release; measures such as infection intensity or explicit consideration of native range parasite burdens might offer more nuanced insight (Kołodziej‐Sobocińska et al. 2018; Prior et al. 2015). However, earlier research of GMPD data suggests that high PSR is linked to higher host mortality in ungulates (Cooper et al. 2012). The extent to which low PSR facilitated invasion and establishment of mammals post‐invasion is an open question signaling a promising area of future research, assuming that appropriate mammal life‐table data becomes available.

Despite the overall decline in PSR, retention of some parasites means there is also potential for the novel weapons hypothesis to play out, depending on spillover processes. Previous work by Marzal et al. (2011) found support for enemy release over novel weapons, while we speculate based on our results that both can happen: net enemy release via the decrease in PSR and novel weapons via the spillover of retained parasites. These processes have the potential to synergistically boost the competitive success of invaders. Lower parasitism might lead to demographic release, and at the same time, retained parasites are most likely to spill over to related hosts with whom the invader already competes (Schatz and Park 2023). Such spillover dynamics, with impacts on native host health, have been observed in several animal‐parasite systems (Strauss et al. 2012; Iglesias et al. 2015; Héritier et al. 2017). For example, native *Emys orbicularis* (pond turtles) in the United Kingdom are known to suffer in competition with invasive *Trachemys scripta elegans* (slider turtles), and researchers have found that spillover of *Spirorchis elegans* blood flukes from *T. s. elegans* is likely exacerbating the decline of *E. orbicularis* (Iglesias et al. 2015; Héritier et al. 2017).

We also found support for the vacated niche hypothesis across our five focal hosts, five parasite types, and four trait dimensions. Lost and acquired parasites showed evidence of taxonomically related replacement based on null model results. Accounting for variation in *z*‐scores, the fact that helminths and arthropods generally have lower *z*‐scores than viruses might be tied to the relative complexity of helminth and arthropod life cycles compared to those of viruses (Pedersen et al. 2005); thus the parasites benefit more or less, respectively, from finding very similar niches. Importantly, the common observation of novel, related parasites colonizing host species that lose parasites suggests that maximal benefits of parasite enemy release are likely to be transient, provided there are related parasites in invaded communities that can exploit the vacated niches.

We also found that lost parasites are generally replaced by acquired parasites with similar phylogenetic specificities. This might suggest that the distribution of parasite specificities is a host trait preserved across ranges; as precedent for this idea, Bordes and Morand (2008) noted that helminth species richness appears to be such a trait as well. A caveat to this is that low sample sizes for some invasions (especially for acquired parasites) could mean that our statistical tests will simply be unable to detect significant differences in specificity distributions, so more data is likely needed before drawing firm conclusions.

Overall, hosts lost and acquired parasites that are relative phylogenetic specialists. The exception is *P. lotor* , which acquired relative generalists among its arthropods, bacteria, and viruses. While this trend only verged on significance, it still raises some interesting observations. *P. lotor* 's invasion is typical as far as enemy release results are concerned, and we still see support for the vacated niche hypothesis from the taxonomic null models (and for parasite type and transmission mode, discussed below). However, the specificity profile of these particular lost and acquired parasites varies slightly. This highlights the general point that exchanging taxonomically related parasites does not necessarily mean those parasites have the same specificity. In a broad sense, this also means that the host will be positioned differently in the overall network of host–parasite associations and might play an unexpected new role as a bridge or reservoir host (Dallas et al. 2019).

The final dimensions we considered for our vacated niche analyses were parasite type and transmission mode. In four of our five focal hosts, we found no significant differences between lost and acquired parasites for these traits. *R. rupicapra* , however, does differ for both traits, presumably due to its total loss of helminths and intermediate transmission parasites with no subsequent acquisition in either category. The non‐acquisition of helminths by this host also meant that we could not include it in taxonomic null model analyses (along with six other focal host–parasite type combinations; missing points in Figure 3). We did find support for vacated niche dynamics in terms of lost and acquired parasite specificities, but lost and acquired parasites with the same distribution of specificities do not necessarily have the same trait profile in other ways.

Our exploration of the vacated niche spanned four areas: parasite taxonomy, phylogenetic specificity, parasite type, and transmission mode. However, there are numerous other dimensions worth considering once sufficient data is available. As noted above for enemy release, measures of infection intensity or direct parasite burden on the host represent an opportunity to quantify parasitism and thus should be incorporated into questions about how host–parasite relationships do or do not change across ranges. In addition, incorporating life stage data for parasites such as helminths has the potential to provide more nuanced results. Researchers might also consider the functional niches of parasites and whether those are conserved between lost and acquired parasites. We might be able to draw on the substantial parasite sharing literature as well (Dallas et al. 2017b; Huang et al. 2014). If a host invades a range in which it is predicted to share parasites with certain native hosts, the parasites of those native hosts might form the pool from which parasites are acquired; researchers could then leverage the trait profile of that parasite pool relative to the invasive host's lost parasites to project how vacated niche dynamics might play out. Additionally, host relatedness or ecological similarity might be used as a predictor of which parasites will fill vacated niches or as a filter to identify parasites of particular interest. Another extension of this work would be to explore what happens when the vacated niche hypothesis is not satisfied and parasite trait profiles shift meaningfully over the course of invasion: what does this mean for future “stepping stone” invasions or range shifts that stem from the new invasive population? Even small changes could have interesting ripple effects, given the population bottlenecks that can occur in some invasions (Ficetola et al. 2008).

We acknowledge limitations in our data and approaches. The GMPD, while extensive and widely used, only included sufficient parasite sampling data for us to consider five focal hosts; broader taxonomic coverage would of course strengthen generalizations drawn from our results. Development of new or improved statistical methods to accommodate low sample sizes would also increase host inclusion in analyses such as ours. Our five focal hosts also all represent invasions facilitated by human translocation of animals (Ikeda et al. 2004; Long 2003; Martínková et al. 2012), so for future work, it would help to have different kinds of range shifts across which to generalize. Climate‐tracking hosts, for example, might retain more of their parasites through connections to source populations or due to gradual parasite adaptation to environmental conditions (Urban 2020). In addition, GMPD sampling effort is uneven across hosts and parasites (Teitelbaum et al. 2020). This is demonstrated, for example, by the paucity of protozoa records available for the taxonomic null model analysis. Despite these shortcomings, the GMPD is a comprehensive database with error‐checking steps included in its assembly (Stephens et al. 2017). It is a vital resource for approaching macroecological questions such as ours.

Our particular definitions of parasite retention and acquisition are also, by necessity, imperfect simplifications of reality. Acquired parasites, for example, might simply have gone unsampled in the focal host's native range, while parasites classified as lost might have been missed in the invaded range. Retained parasites might actually have been lost and then re‐acquired via spillover; alternatively, the association might not persist in the long term. Our previous investigations, however, indicate little to no predictive signal of time since invasion on host–parasite association patterns (Schatz and Park 2023). Accordingly, we generalize our definitions across invasions of different ages. More broadly speaking, it unfortunately is not feasible to account for every possibility and nuance in the complex host–parasite coinvasion process. Our definitions standardize and clearly delineate parasite fates for the purposes of this study, but that does not mean they will be appropriate for, or applicable to, every question.

## 5 Conclusion

With climate change and anthropogenic impacts continuing to impact the planet, it is increasingly urgent that we understand the implications of host redistribution and disease interventions for changing host–parasite associations (Levine and D'Antonio 2003; Carlson et al. 2017). Our work suggests that both the enemy release hypothesis and the vacated niche hypothesis provide valuable frameworks through which to understand and predict such changes. We add greater nuance to enemy release analyses by incorporating specific data on parasite fates—retention or loss, acquisition or non‐acquisition. We also begin to move the vacated niche hypothesis past the anecdotal stage, providing evidence of its generality across five focal hosts, five parasite types, and four trait dimensions. This idea of considering parasite niche space is a useful complement to the host‐centric approach of using host relatedness to predict parasite sharing.

Moving forward, this work has important implications for anticipating invasion trajectories and answering conservation questions. Support for the vacated niche hypothesis, for example, suggests that parasite loss offers information to predict parasite acquisition; this might be useful early on in invasion scenarios to predict future parasite associations and anticipate community‐level impacts of changing parasite circulation. As previously noted, though, we also need more data on parasite pressure and the burdens imposed on hosts for a more holistic understanding of how changing parasite associations directly impact hosts (Kołodziej‐Sobocińska et al. 2018; Prior et al. 2015). Relatedly, research should consider the possibility of transient spikes in parasite virulence during invasions (Bolker et al. 2009); for example, the like‐for‐like parasite exchange predicted by the vacated niche hypothesis does not imply similarity in disease impacts. Overall, the support we find for vacated niches to explain parasite species replacements during species invasions promises to guide assessment of parasite ranges and novel host associations in broader contexts, including the impact of climate change and other anthropogenic influences that are anticipated to perturb ecological communities and ecosystems (Hobbs et al. 2018).

## Author Contributions

**Annakate M. Schatz:** conceptualization (equal), data curation (lead), formal analysis (lead), investigation (lead), methodology (lead), visualization (lead), writing – original draft (lead), writing – review and editing (equal). **Andrew W. Park:** conceptualization (equal), data curation (supporting), formal analysis (supporting), funding acquisition (lead), methodology (supporting), resources (lead), writing – original draft (supporting), writing – review and editing (equal).

## Conflicts of Interest

The authors declare no conflicts of interest.

## Supporting information


Appendix S1.

## Data Availability

Data and reproducible code are available on Dryad (doi: 10.5061/dryad.37pvmcvsp) and Zenodo (doi: 10.5281/zenodo.14756595).

## References

[ece370959-bib-0001] Baron, M. D. , S. Parida , and C. Oura . 2011. “Peste Des Petits Ruminants: A Suitable Candidate for Eradication?” Veterinary Record 169: 16–21. 10.1136/vr.d3947. 21724765

[ece370959-bib-0002] Baselga, A. 2023. “betapart: Partitioning Beta Diversity Into Turnover and Nestedness Components.”

[ece370959-bib-0003] Bashey, F. 2015. “Within‐Host Competitive Interactions as a Mechanism for the Maintenance of Parasite Diversity.” Philosophical Transactions of the Royal Society, B: Biological Sciences 370: 20140301. 10.1098/rstb.2014.0301. PMC4528499 26150667

[ece370959-bib-0004] Bininda‐Emonds, O. R. P. 2007. “The Delayed Rise of Present‐Day Mammals.” Nature 446: 507–512. 10.1038/nature05634. 17392779

[ece370959-bib-0005] Bolker, B. M. , A. Nanda , and D. Shah . 2009. “Transient Virulence of Emerging Pathogens.” Journal of the Royal Society Interface 7, no. 46: 811–822. 10.1098/rsif.2009.0384. 19864267 PMC2874237

[ece370959-bib-0006] Bordes, F. , and S. Morand . 2008. “Helminth Species Diversity of Mammals: Parasite Species Richness Is a Host Species Attribute.” Parasitology 135: 1701–1705. 10.1017/S0031182008005040. 18992179

[ece370959-bib-0007] Callaway, R. M. , and W. M. Ridenour . 2004. “Novel Weapons: Invasive Success and the Evolution of Increased Competitive Ability.” Frontiers in Ecology and the Environment 2: 436–443. 10.1890/1540-9295(2004)002[0436:NWISAT]2.0.CO;2.

[ece370959-bib-0008] Carlson, C. J. , K. R. Burgio , E. R. Dougherty , et al. 2017. “Parasite Biodiversity Faces Extinction and Redistribution in a Changing Climate.” Science Advances 3: e1602422. 10.1126/sciadv.1602422. 28913417 PMC5587099

[ece370959-bib-0009] Chen, I.‐C. , J. K. Hill , R. Ohlemüller , D. B. Roy , and C. D. Thomas . 2011. “Rapid Range Shifts of Species Associated With High Levels of Climate Warming.” Science 333: 1024–1026. 10.1126/science.1206432. 21852500

[ece370959-bib-0010] Cizauskas, C. A. , C. J. Carlson , K. R. Burgio , et al. 2017. “Parasite Vulnerability to Climate Change: An Evidence‐Based Functional Trait Approach.” Royal Society Open Science 4: 160535. 10.1098/rsos.160535. 28280551 PMC5319317

[ece370959-bib-0011] Cooper, N. , J. M. Kamilar , and C. L. Nunn . 2012. “Host Longevity and Parasite Species Richness in Mammals.” PLoS One 7: e42190. 10.1371/journal.pone.0042190. 22879916 PMC3413396

[ece370959-bib-0012] Cumming, G. S. 1999. “Host Distributions Do Not Limit the Species Ranges of Most African Ticks (Acari: Ixodida).” Bulletin of Entomological Research 89: 303–327. 10.1017/S0007485399000450.

[ece370959-bib-0013] Dallas, T. A. , B. A. Han , C. L. Nunn , A. W. Park , P. R. Stephens , and J. M. Drake . 2019. “Host Traits Associated With Species Roles in Parasite Sharing Networks.” Oikos 128: 23–32. 10.1111/oik.05602.

[ece370959-bib-0014] Dallas, T. A. , A. W. Park , and J. M. Drake . 2017a. “Predicting Cryptic Links in Host‐Parasite Networks.” PLoS Computational Biology 13: e1005557. 10.1371/journal.pcbi.1005557. 28542200 PMC5466334

[ece370959-bib-0015] Dallas, T. A. , A. W. Park , and J. M. Drake . 2017b. “Predictability of Helminth Parasite Host Range Using Information on Geography, Host Traits and Parasite Community Structure.” Parasitology 144: 200–205. 10.1017/S0031182016001608. 27762175

[ece370959-bib-0016] Dáttilo, W. , N. Barrozo‐Chávez , A. Lira‐Noriega , et al. 2020. “Species‐Level Drivers of Mammalian Ectoparasite Faunas.” Journal of Animal Ecology 89: 1754–1765. 10.1111/1365-2656.13216. 32198927

[ece370959-bib-0017] Despres, L. , D. Imbert‐Establet , C. Combes , and F. Bonhomme . 1992. “Molecular Evidence Linking Hominid Evolution to Recent Radiation of Schistosomes (Platyhelminthes: Trematoda).” Molecular Phylogenetics and Evolution 1: 295–304. 10.1016/1055-7903(92)90005-2. 1342945

[ece370959-bib-0018] Drost, H.‐G. , A. Gabel , J. Liu , M. Quint , and I. Grosse . 2018. “myTAI: Evolutionary Transcriptomics With R.” Bioinformatics 34: 1589–1590. 10.1093/bioinformatics/btx835. 29309527 PMC5925770

[ece370959-bib-0019] Ficetola, G. F. , A. Bonin , and C. Miaud . 2008. “Population Genetics Reveals Origin and Number of Founders in a Biological Invasion.” Molecular Ecology 17: 773–782. 10.1111/j.1365-294X.2007.03622.x. 18194168

[ece370959-bib-0020] Flory, S. L. , and K. Clay . 2013. “Pathogen Accumulation and Long‐Term Dynamics of Plant Invasions.” Journal of Ecology 101: 607–613. 10.1111/1365-2745.12078.

[ece370959-bib-0021] Gendron, A. D. , D. J. Marcogliese , and M. Thomas . 2011. “Invasive Species Are Less Parasitized Than Native Competitors, but for How Long? The Case of the Round Goby in the Great Lakes‐St. Lawrence Basin.” Biological Invasions 14, no. 2: 367–384. 10.1007/s10530-011-0083-y.

[ece370959-bib-0022] Hellmann, J. J. , J. E. Byers , B. G. Bierwagen , and J. S. Dukes . 2008. “Five Potential Consequences of Climate Change for Invasive Species.” Conservation Biology 22: 534–543. 10.1111/j.1523-1739.2008.00951.x. 18577082

[ece370959-bib-0023] Héritier, L. , A. Valdeón , A. Sadaoui , et al. 2017. “Introduction and Invasion of the Red‐Eared Slider and Its Parasites in Freshwater Ecosystems of Southern Europe: Risk Assessment for the European Pond Turtle in Wild Environments.” Biodiversity and Conservation 26: 1817–1843. 10.1007/s10531-017-1331-y.

[ece370959-bib-0024] Herrera, J. P. , D. Chakraborty , J. Rushmore , S. Altizer , and C. Nunn . 2019. “The Changing Ecology of Primate Parasites: Insights From Wild‐Captive Comparisons.” American Journal of Primatology 81: e22991. 10.1002/ajp.22991. 31265141

[ece370959-bib-0025] Hijmans, R. J. , S. E. Cameron , J. L. Parra , P. G. Jones , and A. Jarvis . 2005. “Very High Resolution Interpolated Climate Surfaces for Global Land Areas.” International Journal of Climatology 25: 1965–1978. 10.1002/joc.1276.

[ece370959-bib-0026] Hobbs, R. J. , L. E. Valentine , R. J. Standish , and S. T. Jackson . 2018. “Movers and Stayers: Novel Assemblages in Changing Environments.” Trends in Ecology & Evolution 33: 116–128. 10.1016/j.tree.2017.11.001. 29173900

[ece370959-bib-0027] Holt, B. G. 2013. “An Update of Wallace's Zoogeographic Regions of the World.” Science 339: 74–78. 10.1126/science.1228282. 23258408

[ece370959-bib-0028] Huang, S. , O. R. P. Bininda‐Emonds , P. R. Stephens , J. L. Gittleman , and S. Altizer . 2014. “Phylogenetically Related and Ecologically Similar Carnivores Harbour Similar Parasite Assemblages.” Journal of Animal Ecology 83: 671–680. 10.1111/1365-2656.12160. 24289314

[ece370959-bib-0029] Iglesias, R. , J. M. García‐Estévez , C. Ayres , A. Acuña , and A. Cordero‐Rivera . 2015. “First Reported Outbreak of Severe Spirorchiidiasis in *Emys orbicularis*, Probably Resulting From a Parasite Spillover Event.” Diseases of Aquatic Organisms 113, no. 1: 75–80. 10.3354/dao02812. 25667339

[ece370959-bib-0030] Ikeda, T. , M. Asano , Y. Matoba , and G. Abe . 2004. “Present Status of Invasive Alien Raccoon and Its Impact in Japan.” Global Environmental Research 8: 125–131.

[ece370959-bib-0031] IUCN . 2016. “The IUCN Red List of Threatened Species, Version 2016‐1.” https://www.iucnredlist.org/.

[ece370959-bib-0032] Johnson, D. S. , J. D. Shields , D. Doucette , and R. Heard . 2020. “A Climate Migrant Escapes Its Parasites.” Marine Ecology Progress Series 641: 111–121. 10.3354/meps13278.

[ece370959-bib-0033] Johnson, P. T. J. , J. C. Roode , and A. Fenton . 2015. “Why Infectious Disease Research Needs Community Ecology.” Science 349: 1259504. 10.1126/science.1259504. 26339035 PMC4863701

[ece370959-bib-0034] Jones, K. E. , J. Bielby , M. Cardillo , et al. 2009. “PanTHERIA: A Species‐Level Database of Life History, Ecology, and Geography of Extant and Recently Extinct Mammals.” Ecology 90: 2648. 10.1890/08-1494.1.

[ece370959-bib-0035] Kelly, D. W. , R. A. Paterson , C. R. Townsend , R. Poulin , and D. M. Tompkins . 2009. “Parasite Spillback: A Neglected Concept in Invasion Ecology?” Ecology 90: 2047–2056. 10.1890/08-1085.1. 19739367

[ece370959-bib-0036] Kembel, S. W. , P. D. Cowan , M. R. Helmus , et al. 2010. “Picante: R Tools for Integrating Phylogenies and Ecology.” Bioinformatics 26: 1463–1464. 10.1093/bioinformatics/btq166. 20395285

[ece370959-bib-0037] Kołodziej‐Sobocińska, M. , M. Brzeziński , A. Niemczynowicz , and A. Zalewski . 2018. “High Parasite Infection Level in Non‐Native Invasive Species: It Is Just a Matter of Time.” Ecography 41: 1283–1294. 10.1111/ecog.03362.

[ece370959-bib-0038] Lembo, T. , C. Oura , S. Parida , et al. 2013. “Peste Des Petits Ruminants Infection Among Cattle and Wildlife in Northern Tanzania.” Emerging Infectious Diseases 19: 2037–2040. 10.3201/eid1912.130973. 24274684 PMC3840886

[ece370959-bib-0039] Levine, J. M. , and C. M. D'Antonio . 2003. “Forecasting Biological Invasions With Increasing International Trade.” Conservation Biology 17, no. 1: 322–326. 10.1046/j.1523-1739.2003.02038.x.

[ece370959-bib-0040] Lira‐Noriega, A. , and A. T. Peterson . 2014. “Range‐Wide Ecological Niche Comparisons of Parasite, Hosts and Dispersers in a Vector‐Borne Plant Parasite System.” Journal of Biogeography 41: 1664–1673.

[ece370959-bib-0041] Lloyd‐Smith, J. O. 2013. “Vacated Niches, Competitive Release and the Community Ecology of Pathogen Eradication.” Philosophical Transactions of the Royal Society B 368: 20120150. 10.1098/rstb.2012.0150. PMC3720048 23798698

[ece370959-bib-0042] Lloyd‐Smith, J. O. , D. George , K. M. Pepin , et al. 2009. “Epidemic Dynamics at the Human–Animal Interface.” Science 326: 1362–1367. 10.1126/science.1177345. 19965751 PMC3891603

[ece370959-bib-0043] Long, J. L. 2003. Introduced Mammals of the World: Their History, Distribution and Influence. CABI Publishing.

[ece370959-bib-0044] Lymbery, A. J. , M. Morine , H. G. Kanani , S. J. Beatty , and D. L. Morgan . 2014. “Co‐Invaders: The Effects of Alien Parasites on Native Hosts.” International Journal for Parasitology: Parasites and Wildlife 3: 171–177. 10.1016/j.ijppaw.2014.04.002. 25180161 PMC4145144

[ece370959-bib-0045] Maher, S. P. , C. Ellis , K. L. Gage , R. E. Enscore , and A. T. Peterson . 2010. “Range‐Wide Determinants of Plague Distribution in North America.” American Society of Tropical Medicine and Hygiene 83, no. 4: 736–742. 10.4269/ajtmh.2010.10-0042. PMC2946734 20889857

[ece370959-bib-0046] Mainka, S. A. , and G. W. Howard . 2010. “Climate Change and Invasive Species: Double Jeopardy.” Integrative Zoology 5: 102–111. 10.1111/j.1749-4877.2010.00193.x. 21392328

[ece370959-bib-0047] Marr, S. R. , W. J. Mautz , and A. H. Hara . 2008. “Parasite Loss and Introduced Species: A Comparison of the Parasites of the Puerto Rican Tree Frog, (*Eleutherodactylus coqui*), in Its Native and Introduced Ranges.” Biological Invasions 10: 1289–1298. 10.1007/s10530-007-9203-0.

[ece370959-bib-0048] Martin, P. R. , and C. K. Ghalambor . 2023. “A Case for the “Competitive Exclusion–Tolerance Rule” as a General Cause of Species Turnover Along Environmental Gradients.” American Naturalist 202: 1–17. 10.1086/724683. 37384767

[ece370959-bib-0049] Martínková, N. , B. Zemanová , A. Kranz , M. D. Giménez , and P. Hájková . 2012. “Chamois Introductions to Central Europe and New Zealand.” Folia Zoologica 61: 239–245. 10.25225/fozo.v61.i3.a8.2012.

[ece370959-bib-0050] Marzal, A. , R. E. Ricklefs , G. Valkiūnas , et al. 2011. “Diversity, Loss, and Gain of Malaria Parasites in a Globally Invasive Bird.” PLoS One 6: e21905. 10.1371/journal.pone.0021905. 21779353 PMC3136938

[ece370959-bib-0051] Mideo, N. 2009. “Parasite Adaptations to Within‐Host Competition.” Trends in Parasitology 25: 261–268. 10.1016/j.pt.2009.03.001. 19409846

[ece370959-bib-0052] Miller, M. A. , J. M. Kinsella , R. W. Snow , et al. 2020. “Highly Competent Native Snake Hosts Extend the Range of an Introduced Parasite Beyond Its Invasive Burmese Python Host.” Ecosphere 11, no. 6: e03153. 10.1002/ecs2.3153.

[ece370959-bib-0053] Mitchell, C. E. , and A. G. Power . 2003. “Release of Invasive Plants From Fungal and Viral Pathogens.” Nature 421: 625–627. 10.1038/nature01317. 12571594

[ece370959-bib-0054] Molyneux, D. H. , E. Mitre , M. J. Bockarie , and L. A. Kelly‐Hope . 2014. “Filaria Zoogeography in Africa: Ecology, Competitive Exclusion, and Public Health Relevance.” Trends in Parasitology 30: 163–169. 10.1016/j.pt.2014.02.002. 24636357

[ece370959-bib-0055] Morales‐Castilla, I. , P. Pappalardo , M. J. Farrell , et al. 2021. “Forecasting Parasite Sharing Under Climate Change.” Philosophical Transactions of the Royal Society B 376: 20200360. 10.1098/rstb.2020.0360. PMC8450630 34538143

[ece370959-bib-0056] Morand, S. , and R. Poulin . 2003. “Phylogenies, the Comparative Method and Parasite Evolutionary Ecology.” In Advances in Parasitology, 281–302. Academic Press. 10.1016/S0065-308X(03)54006-4. 14711088

[ece370959-bib-0057] Oksanen, J. 2022. “vegan: Community Ecology Package.”

[ece370959-bib-0058] Park, A. W. , M. J. Farrell , J. P. Schmidt , et al. 2018. “Characterizing the Phylogenetic Specialism–Generalism Spectrum of Mammal Parasites.” Proceedings of the Royal Society B: Biological Sciences 285: 20172613. 10.1098/rspb.2017.2613. PMC5879627 29514973

[ece370959-bib-0059] Pedersen, A. B. , S. Altizer , M. Poss , A. A. Cunningham , and C. L. Nunn . 2005. “Patterns of Host Specificity and Transmission Among Parasites of Wild Primates.” International Journal for Parasitology 35: 647–657. 10.1016/j.ijpara.2005.01.005. 15862578

[ece370959-bib-0060] Pedersen, A. B. , and A. Fenton . 2007. “Emphasizing the Ecology in Parasite Community Ecology.” Trends in Ecology & Evolution 22: 133–139. 10.1016/j.tree.2006.11.005. 17137676

[ece370959-bib-0061] Poulin, R. 2005. “Relative Infection Levels and Taxonomic Distances Among the Host Species Used by a Parasite: Insights Into Parasite Specialization.” Parasitology 130: 109–115. 10.1017/S0031182004006304. 15700762

[ece370959-bib-0062] Prior, K. M. , T. H. Q. Powell , A. L. Joseph , and J. J. Hellmann . 2015. “Insights From Community Ecology Into the Role of Enemy Release in Causing Invasion Success: The Importance of Native Enemy Effects.” Biological Invasions 17: 1283–1297. 10.1007/s10530-014-0800-4.

[ece370959-bib-0063] R Development Core Team . 2008. R: A Language and Environment for Statistical Computing. R Development Core Team.

[ece370959-bib-0064] Revell, L. J. 2012. “Phytools: An R Package for Phylogenetic Comparative Biology (and Other Things).” Methods in Ecology and Evolution 3: 217–223. 10.1111/j.2041-210X.2011.00169.x.

[ece370959-bib-0065] Reynolds, M. G. , D. S. Carroll , and K. L. Karem . 2012. “Factors Affecting the Likelihood of Monkeypox's Emergence and Spread in the Post‐Smallpox Era.” Current Opinion in Virology 2: 335–343. 10.1016/j.coviro.2012.02.004. 22709519 PMC9533834

[ece370959-bib-0066] Roeder, P. , J. Mariner , and R. Kock . 2013. “Rinderpest: The Veterinary Perspective on Eradication.” Philosophical Transactions of the Royal Society, B: Biological Sciences 368: 20120139. 10.1098/rstb.2012.0139. PMC3720037 23798687

[ece370959-bib-0067] Schatz, A. M. , and A. W. Park . 2021. “Host and Parasite Traits Predict Cross‐Species Parasite Acquisition by Introduced Mammals.” Proceedings of the Royal Society B: Biological Sciences 288: rspb.2021.0341. 10.1098/rspb.2021.0341. PMC8097221 33947240

[ece370959-bib-0068] Schatz, A. M. , and A. W. Park . 2023. “Patterns of Host–Parasite Coinvasion Promote Enemy Release and Specialist Parasite Spillover.” Journal of Animal Ecology 92: 1029–1041. 10.1111/1365-2656.13910. 36934311

[ece370959-bib-0069] Stephens, P. R. , S. Altizer , K. F. Smith , et al. 2016. “The Macroecology of Infectious Diseases: A New Perspective on Global‐Scale Drivers of Pathogen Distributions and Impacts.” Ecology Letters 19: 1159–1171. 10.1111/ele.12644. 27353433

[ece370959-bib-0070] Stephens, P. R. , P. Pappalardo , S. Huang , et al. 2017. “Global Mammal Parasite Database Version 2.0.” Ecology 98: 1476. 10.1002/ecy.1799. 28273333

[ece370959-bib-0071] Strauss, A. , A. White , and M. Boots . 2012. “Invading With Biological Weapons: The Importance of Disease‐Mediated Invasions.” Functional Ecology 26: 1249–1261. 10.1111/1365-2435.12011.

[ece370959-bib-0072] Teitelbaum, C. S. , C. R. Amoroso , S. Huang , et al. 2020. “A Comparison of Diversity Estimators Applied to a Database of Host–Parasite Associations.” Ecography 43: 1316–1328. 10.1111/ecog.05143.

[ece370959-bib-0073] Torchin, M. E. , K. D. Lafferty , A. P. Dobson , V. J. McKenzie , and A. M. Kuris . 2003. “Introduced Species and Their Missing Parasites.” Nature 421: 628–630. 10.1038/nature01346. 12571595

[ece370959-bib-0074] Torchin, M. E. , K. D. Lafferty , and A. M. Kuris . 2001. “Release From Parasites as Natural Enemies: Increased Performance of a Globally Introduced Marine Crab.” Biological Invasions 3: 333–345. 10.1023/A:1015855019360.

[ece370959-bib-0075] Urban, M. C. 2020. “Climate‐Tracking Species Are Not Invasive.” Nature Climate Change 10: 382–384. 10.1038/s41558-020-0770-8.

[ece370959-bib-0076] Vilcinskas, A. 2015. “Pathogens as Biological Weapons of Invasive Species.” PLoS Pathogens 11: e1004714. 10.1371/journal.ppat.1004714. 25856550 PMC4391917

[ece370959-bib-0077] Weyer, J. , and L. H. Blumberg . 2022. “Monkeypox: Is the ‘Vacated Niche’ Being Filled?” Southern African Journal of Infectious Diseases 37, no. 1: a479. 10.4102/sajid.v37i1.479. PMC9772718 36568331

