# Supplementary material for: Evidence for the Vacated Niche Hypothesis in Parasites of Invasive Mammals
Source: Ecol Evol. 2025 Feb 10;15(2):e70959. doi: 10.1002/ece3.70959 (PMC11808211; doi:10.1002/ece3.70959)
Supplement: Supplementary file 1 — Appendix S1. [file ECE3-15-e70959-s001.pdf]

Appendix for "Evidence for the vacated niche hypothesis in parasites of invasive mammals"

Annakate M. Schatz[1,2,*] (schat22a@mtholyoke.edu) and Andrew W. Park[1,2,3] (awpark@uga.edu)
[1] Odum School of Ecology, [2] Center for the Ecology of Infectious Diseases, [3] Department of Infectious Diseases, College of Veterinary Medicine, University of Georgia
* Current affiliation: Foreign Animal Disease Research Unit, USDA-ARS, National Bio and Agro-Defense Facility, Manhattan, KS 66506, USA

(a) *Cervus elaphus*

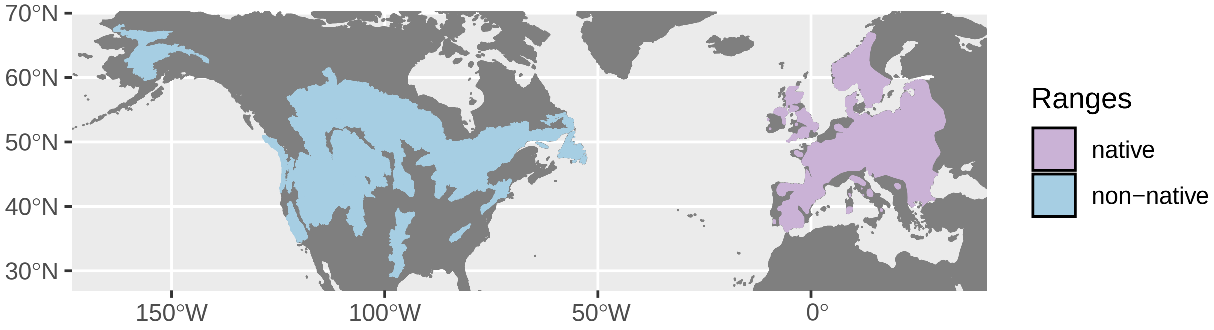

(b) *Procyon lotor*

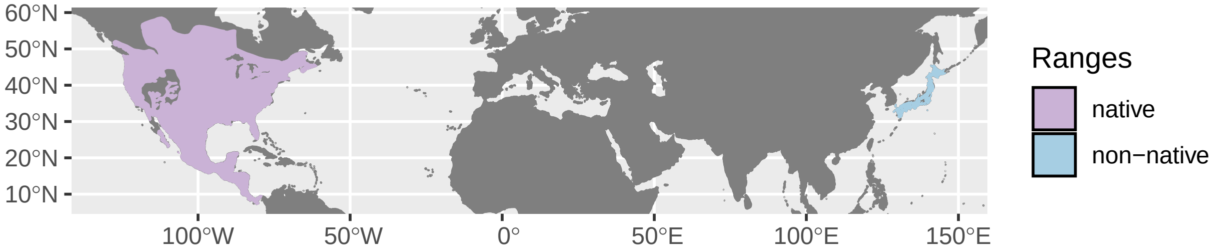

(c) *Rupicapra rupicapra*

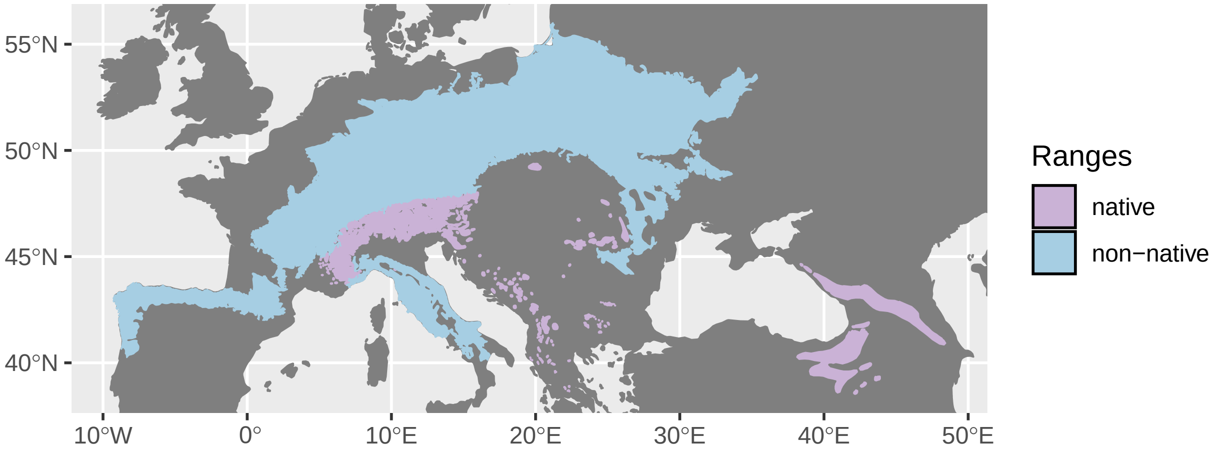

(d) *Sus scrofa*

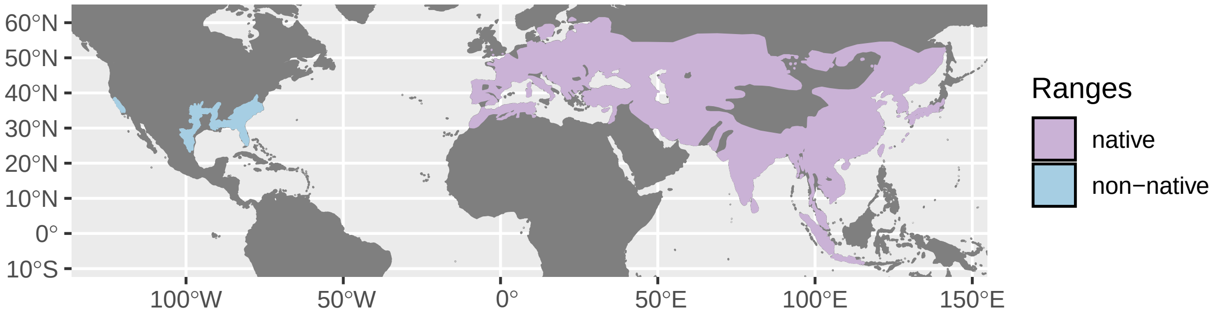

(e) *Vulpes vulpes*

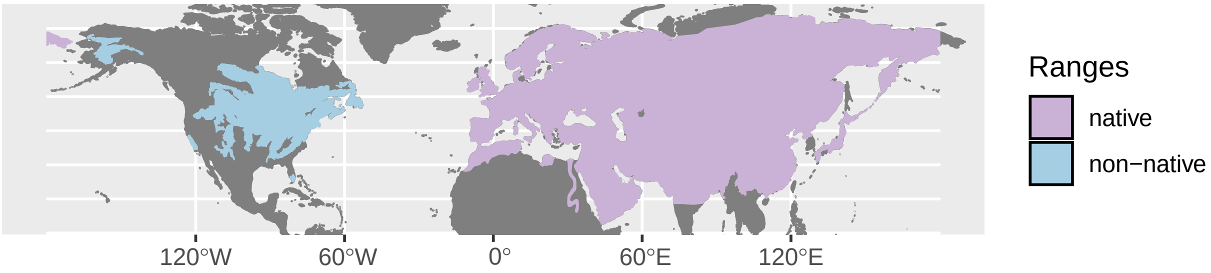

Figure A1. Range maps developed for each focal host: (a) *Cervus elaphus* (red deer), (b) *Procyon lotor* (common raccoon), (c) *Rupicapra rupicapra* (chamois), (d) *Sus scrofa* (wild boar), and (e) *Vulpes vulpes* (red fox). For map clarity, un-buffered IUCN native ranges are shown. Only non-native ranges included in our analysis are shown.

(a) *Cervus elaphus*

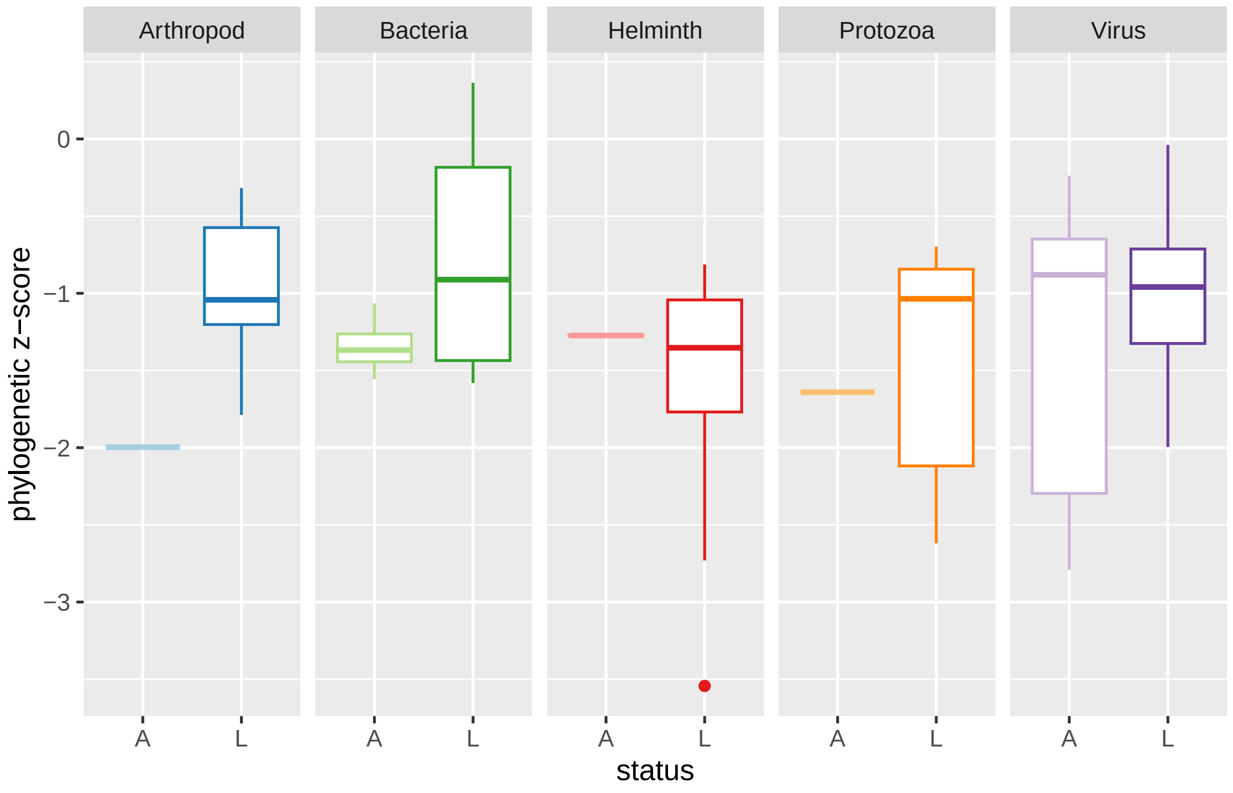

(b) *Procyon lotor*

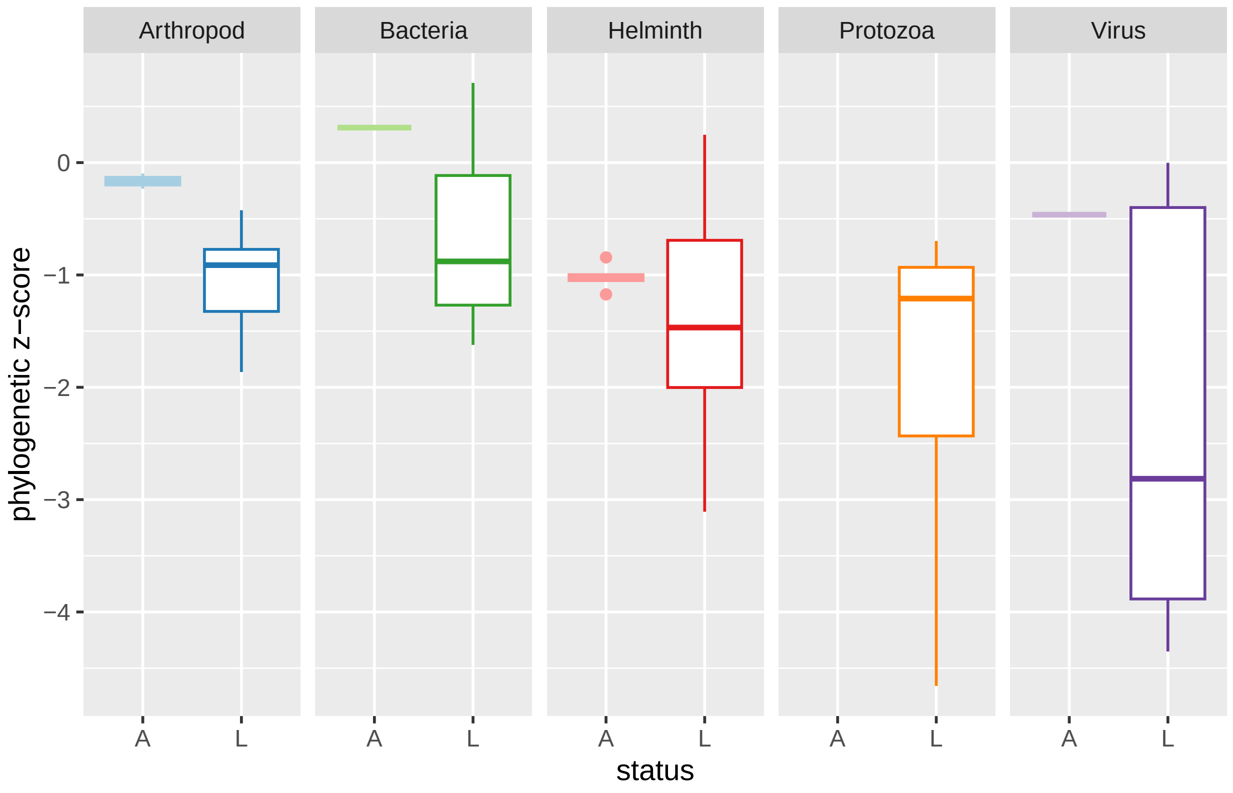

(c) *Rupicapra rupicapra*

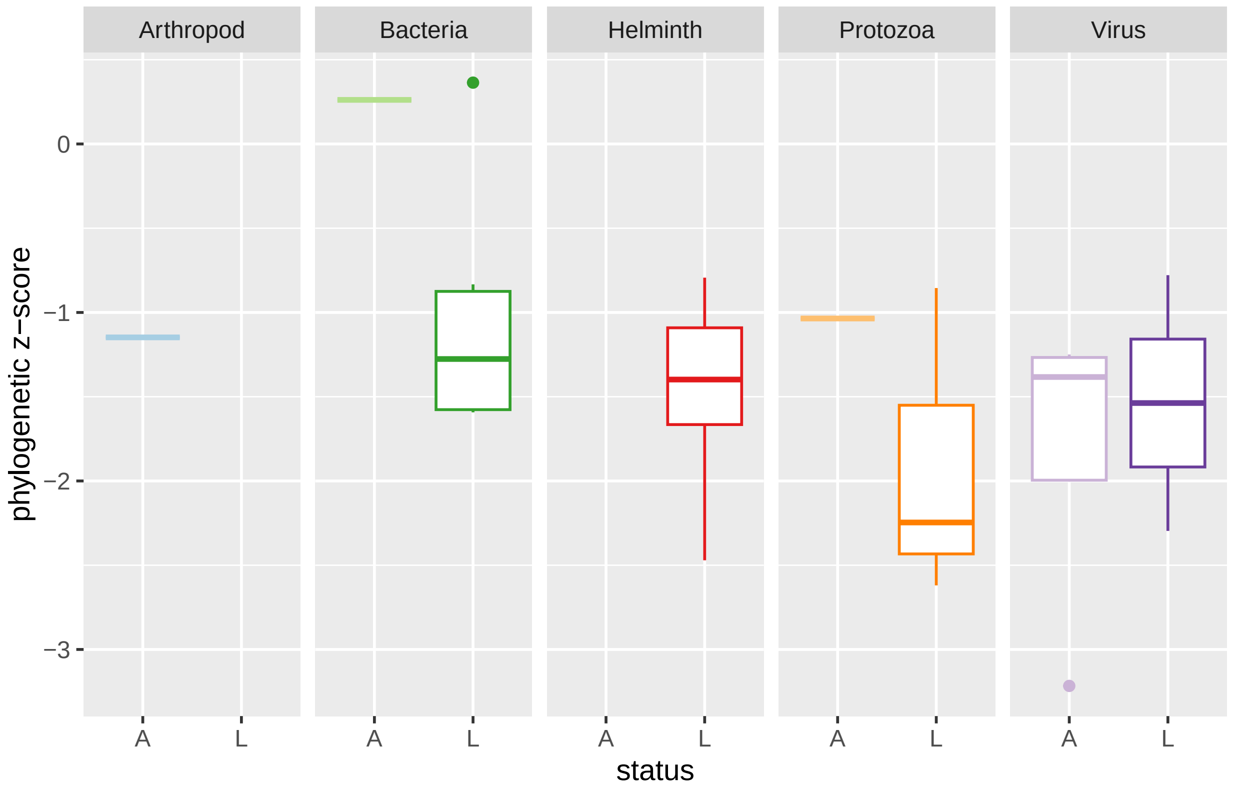

(d) *Sus scrofa*

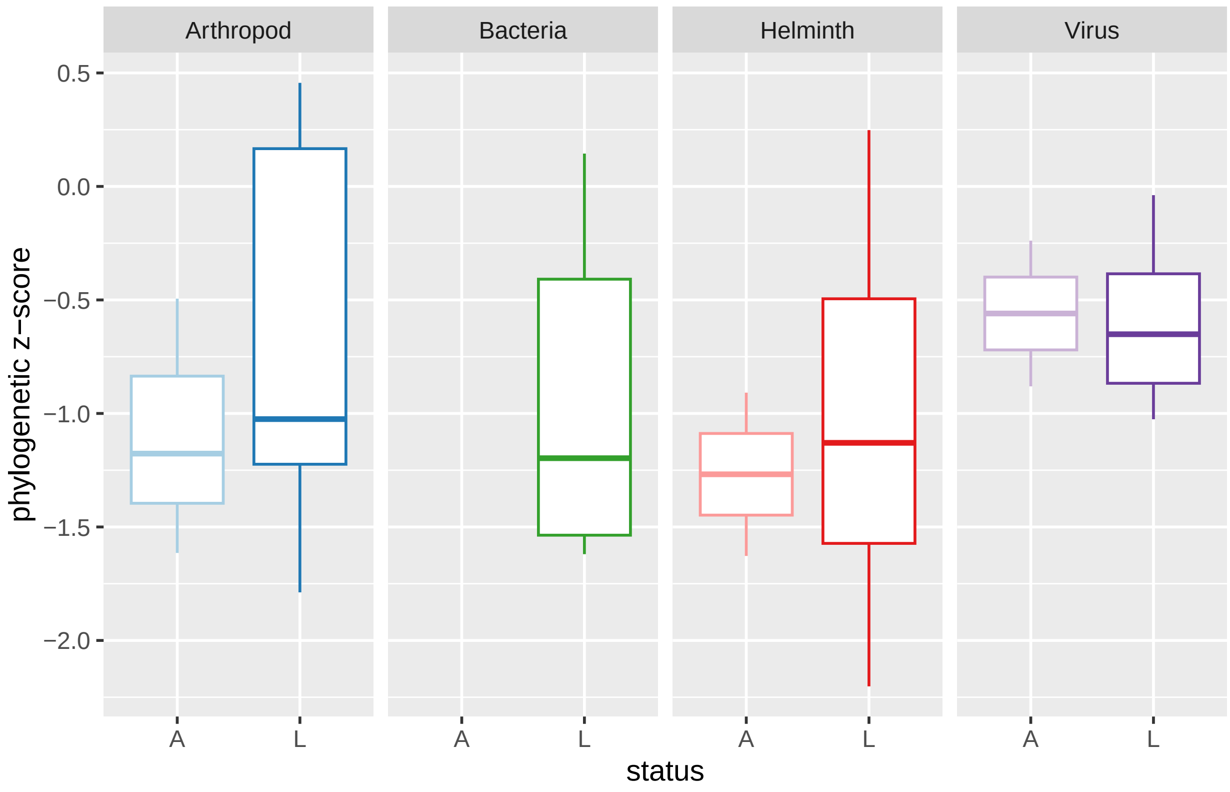

Arthropod
Bacteria
Helminth
Virus
0.5
0.0
−0.5
−1.0
−1.5
−2.0
phylogenetic z−score
A
L
status

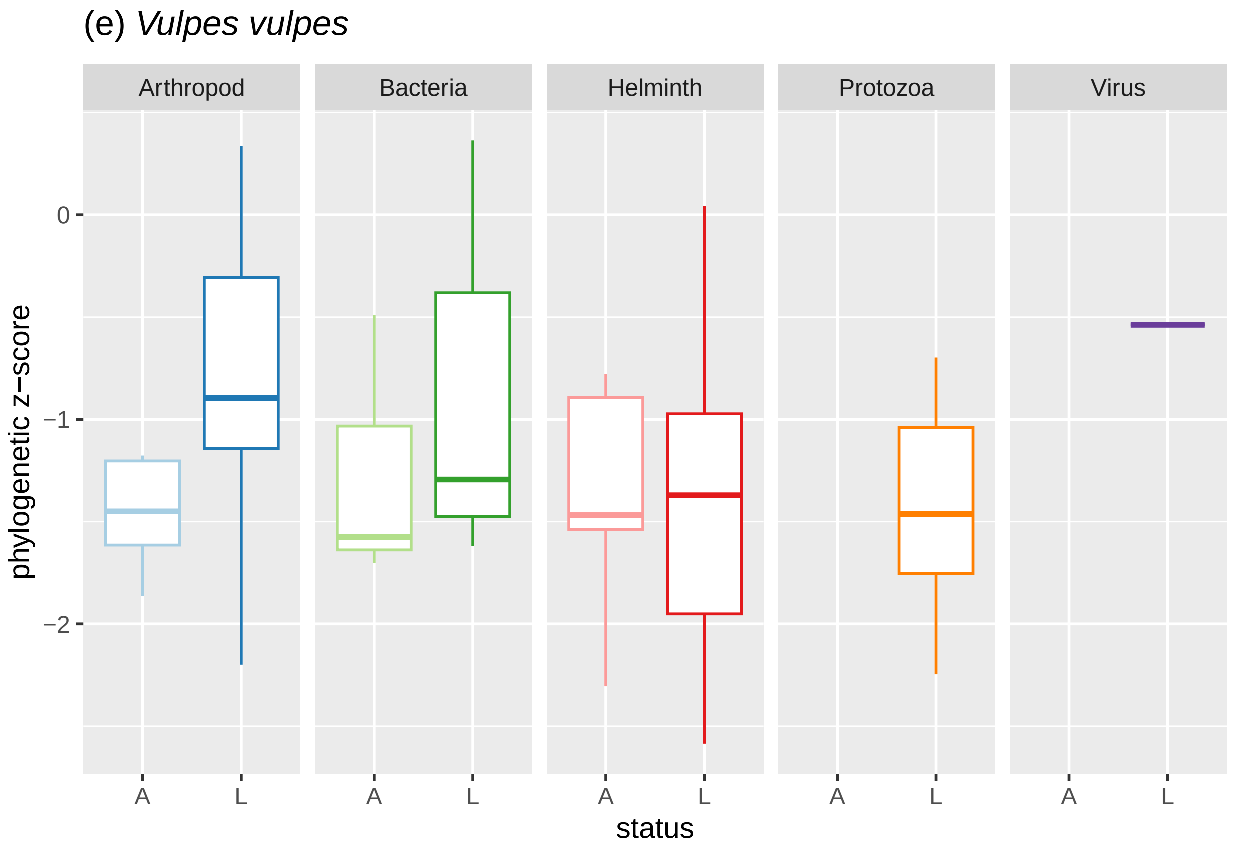

Figure A2. Boxplots of phylogenetic host specificities for acquired (A) and lost (L) parasites for each focal host, grouped by parasite type.

(a) *Cervus elaphus*

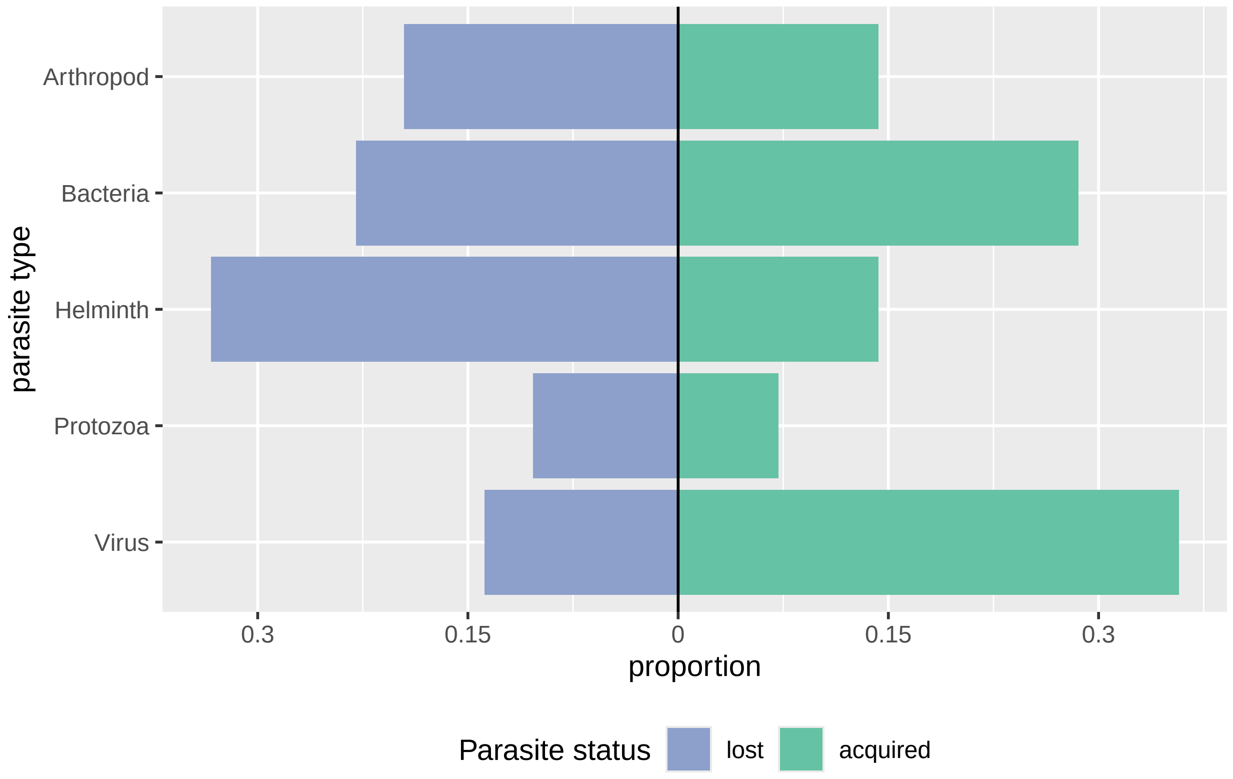

(b) *Procyon lotor*

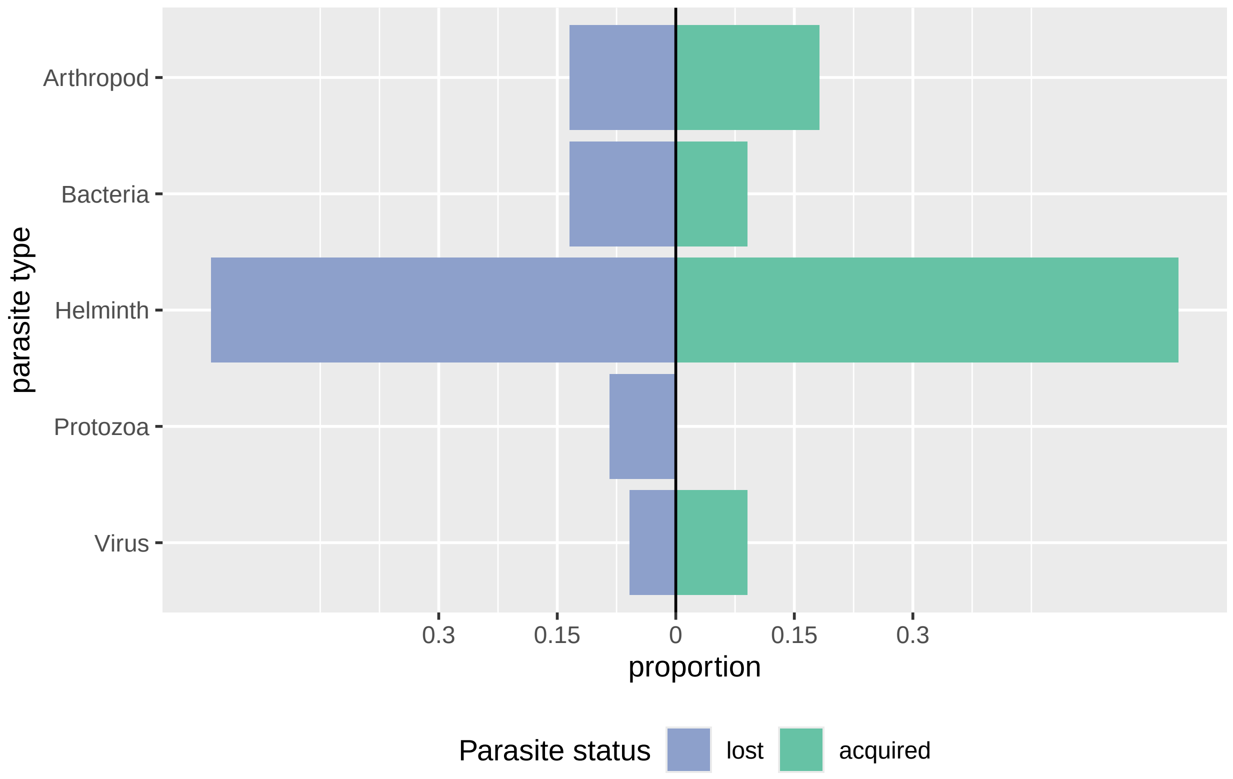

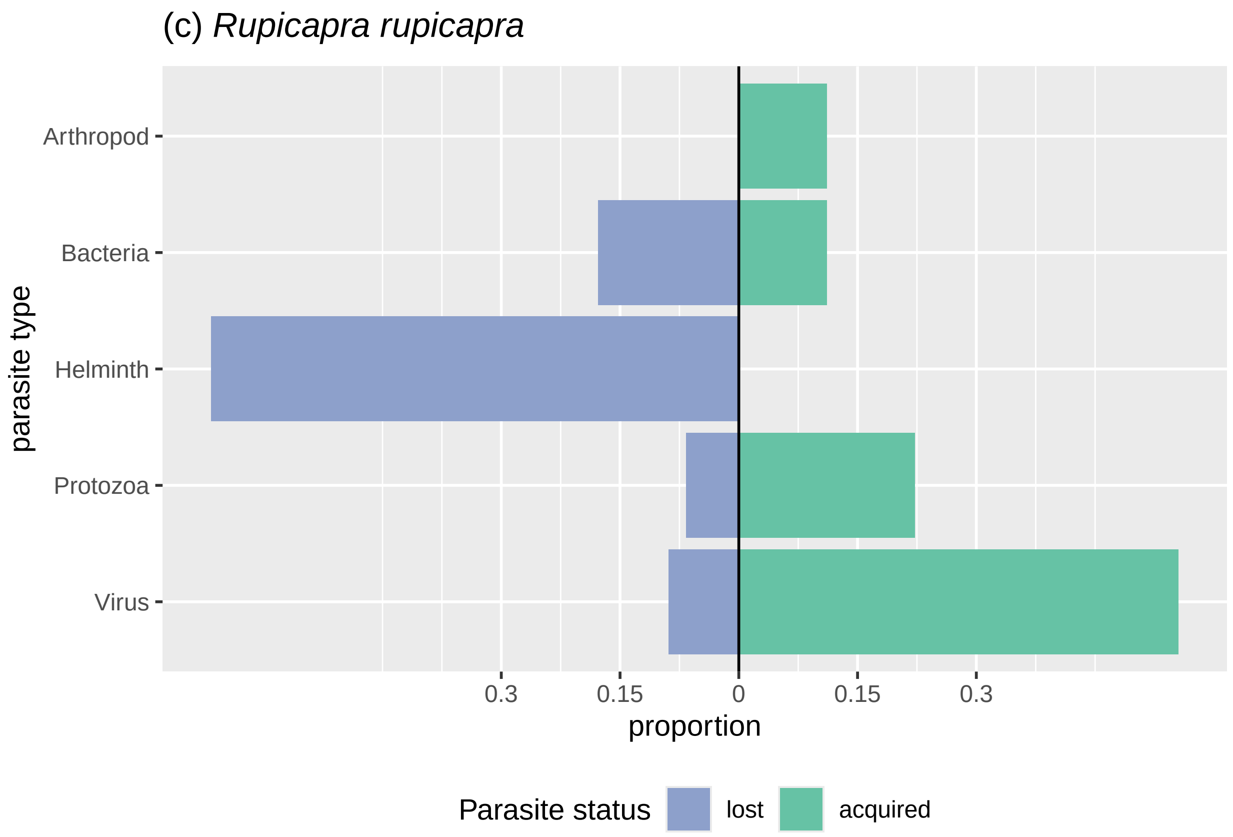
(c) Rupicapra rupicapra
parasite type
Arthropod
Bacteria
Helminth
Protozoa
Virus
0.3
0.15
0
0.15
0.3
proportion
Parasite status
lost
acquired

(d) *Sus scrofa*

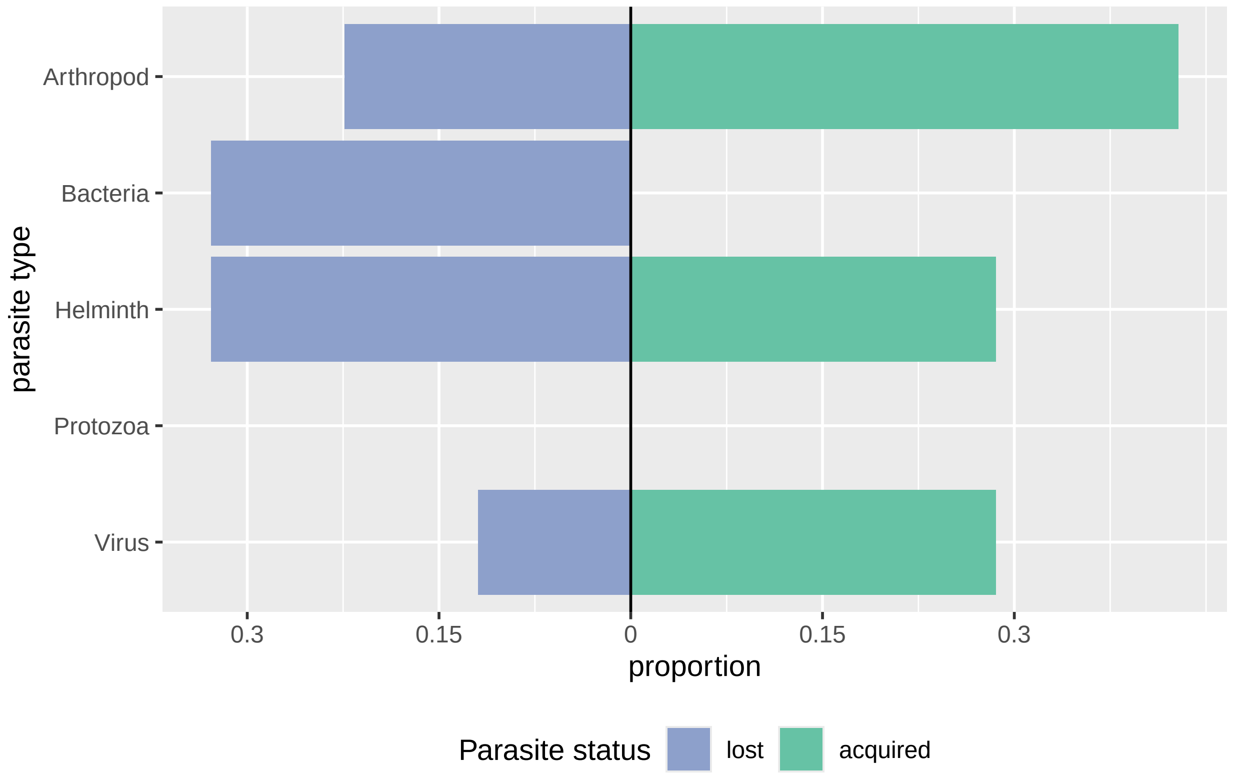

(e) *Vulpes vulpes*

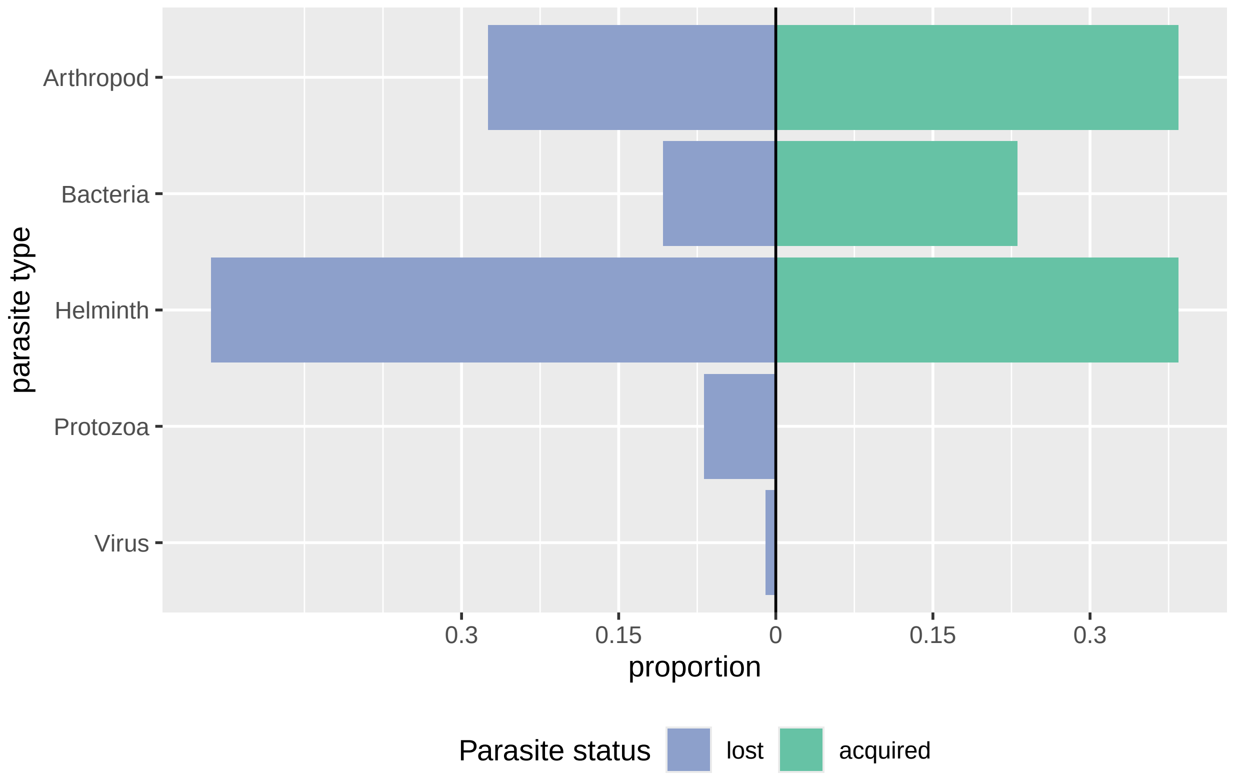

Figure A3. Distribution of lost and acquired parasite types for each focal host. Denominator for proportions is total lost parasites (left panels) and total acquired parasites (right panels) summed across parasite types.

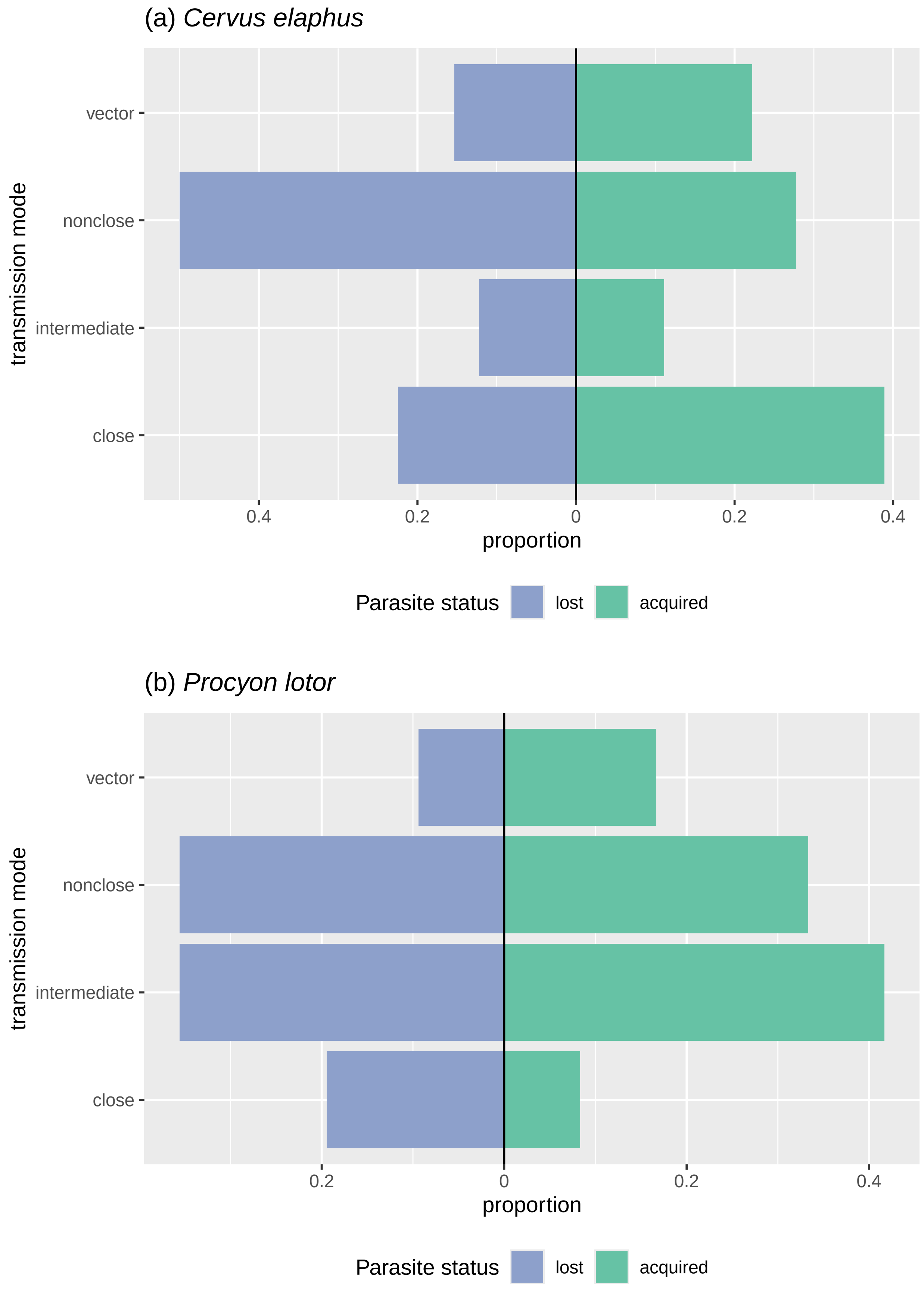

(a) *Cervus elaphus*
vector
nonclose
intermediate
close
transmission mode
0.4
0.2
0
0.2
0.4
proportion
Parasite status
lost
acquired
(b) *Procyon lotor*
vector
nonclose
intermediate
close
transmission mode
0.2
0
0.2
0.4
proportion
Parasite status
lost
acquired

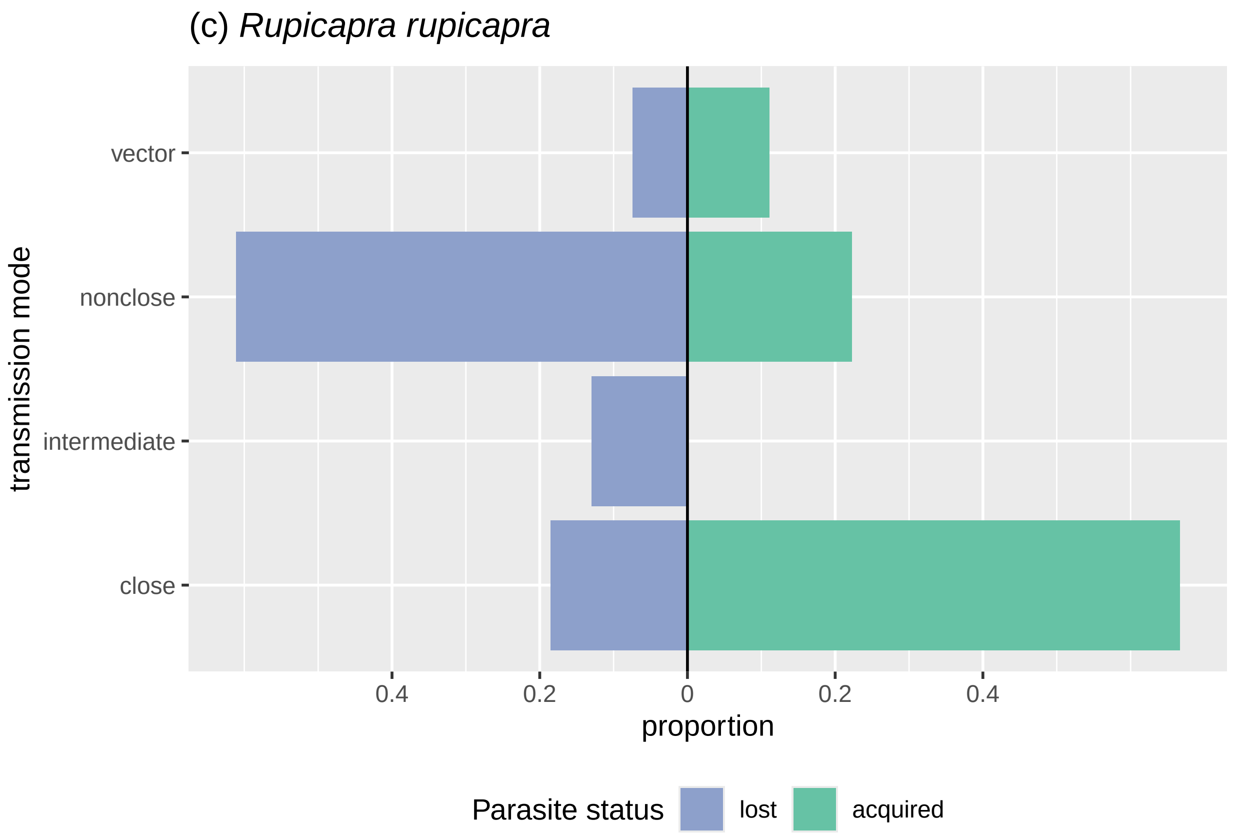
(c) Rupicapra rupicapra
vector
nonclose
intermediate
close
transmission mode
0.4
0.2
0
0.2
0.4
proportion
Parasite status
lost
acquired

(d) *Sus scrofa*

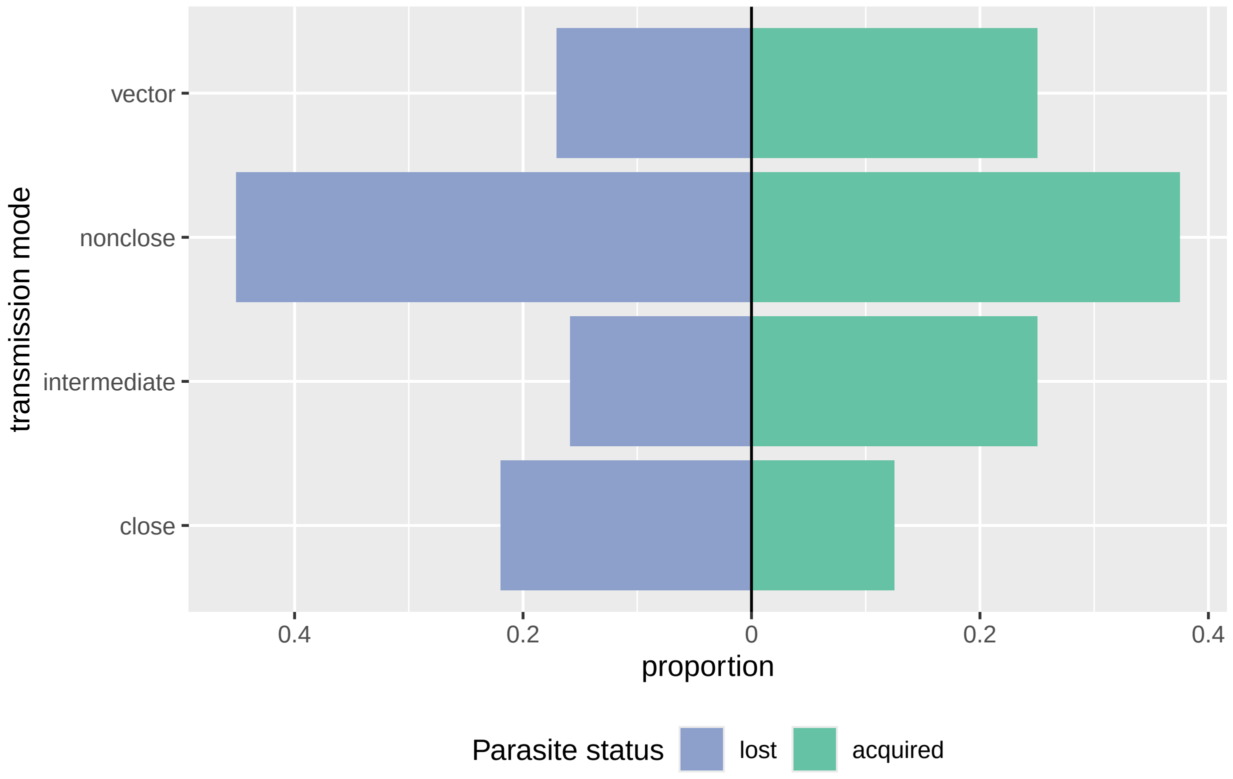

(e) *Vulpes vulpes*

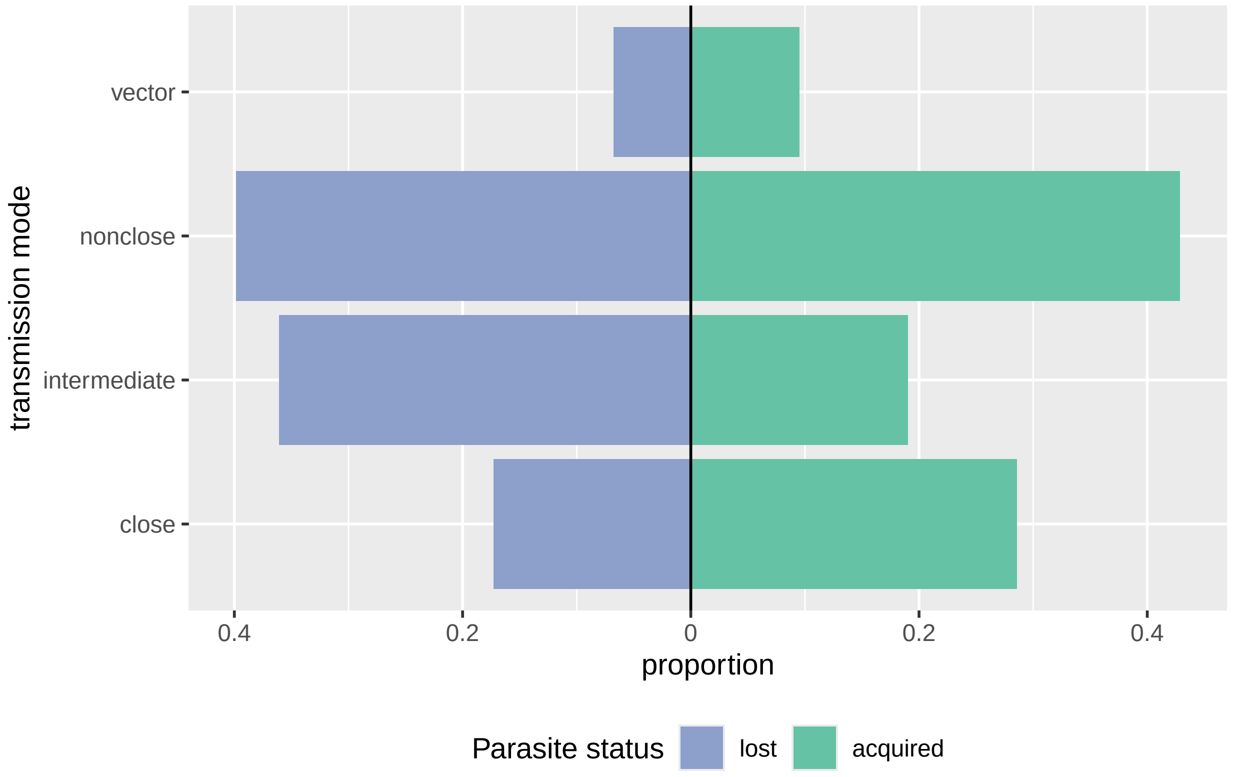

Figure A4. Distribution of lost and acquired parasite transmission modes for each focal host. Denominator for proportions is total lost parasites (left panels) and total acquired parasites (right panels) summed across transmission modes.

Table A1. Summary of GMPD sampling data available for each focal host, structured by parasite status (A = acquired, L = lost, R = retained, notA = not acquired). Mean publications calculated as average number of unique references per host-parasite pair within a given status. Mean animals calculated as average number of animals sampled per host-parasite pair within a given status. Mean values rounded to one decimal place.

Focal host	Parasite status	Mean publications	Mean animals
Cervus elaphus	A	1.8	706.1
Cervus elaphus	L	1.9	281.1
Cervus elaphus	R	5.5	917.4
Cervus elaphus	notA	3.2	595.9
Procyon lotor	A	1.4	597.4
Procyon lotor	L	2.6	711.6
Procyon lotor	R	3.7	1549.6
Procyon lotor	notA	3.6	1143.6
Rupicapra rupicapra	A	1.0	61.0
Rupicapra rupicapra	L	1.9	254.5
Rupicapra rupicapra	R	3.4	283.0
Rupicapra rupicapra	notA	2.7	296.0
Sus scrofa	A	1.1	873.0
Sus scrofa	L	1.5	1270.6
Sus scrofa	R	4.6	5947.2
Sus scrofa	notA	2.8	3729.3
Vulpes vulpes	A	1.2	60.2
Vulpes vulpes	L	2.6	885.0
Vulpes vulpes	R	12.8	7886.0
Vulpes vulpes	notA	8.5	4763.8

Table A2. Summary of parasite data for each focal host included in this study. Parasite types (arthropod, bacteria, helminth, protozoa, virus) summarized at family level, with species counts per family listed under the respective host from which they were sampled (including native and non-native ranges).

**Parasite**		**Focal host**				
Type	Family	*Cervus elaphus*	*Procyon lotor*	*Rupicapra rupicapra*	*Sus scrofa*	*Vulpes vulpes*
Arthropod	Ceratophyllidae	NA	1	NA	NA	3
Arthropod	Demodecidae	NA	NA	NA	1	NA
Arthropod	Demodicidae	1	NA	NA	NA	NA
Arthropod	Hippoboscidae	NA	NA	NA	NA	2
Arthropod	Ixodidae	12	10	1	17	16
Arthropod	Laelapidae	NA	1	NA	NA	NA
Arthropod	Linguatulidae	NA	NA	NA	NA	1
Arthropod	Macronyssidae	NA	1	NA	NA	NA
Arthropod	Muscidae	1	NA	NA	NA	NA
Arthropod	Oestridae	4	2	NA	NA	NA
Arthropod	Pinnotheridae	NA	NA	NA	NA	1
Arthropod	Psoroptidae	NA	NA	NA	NA	1
Arthropod	Pulicidae	NA	1	NA	NA	8
Arthropod	Sarcoptidae	1	NA	1	NA	1
Arthropod	Trichodectidae	NA	1	NA	NA	NA
Arthropod	Vermipsyllidae	NA	1	NA	NA	2
Bacteria	Actinomycetaceae	1	NA	NA	NA	NA
Bacteria	Anaplasmataceae	2	4	1	2	2
Bacteria	Bartonellaceae	2	1	NA	NA	1
Bacteria	Borreliaceae	1	1	1	1	2
Bacteria	Brucellaceae	1	NA	1	1	2
Bacteria	Chlamydiaceae	2	NA	1	1	NA
Bacteria	Coxiellaceae	1	1	1	1	NA
Bacteria	Dermatophilaceae	NA	NA	1	NA	NA
Bacteria	Desulfovibrionaceae	1	NA	NA	NA	NA
Bacteria	Enterobacteriaceae	2	2	2	NA	1
Bacteria	Enterococcaceae	NA	1	NA	NA	NA
Bacteria	Erysipelotrichaceae	NA	NA	NA	1	NA

Bacteria	Francisellaceae	1	1	NA	1	NA
Bacteria	Leptospiraceae	1	4	NA	1	1
Bacteria	Listeriaceae	NA	NA	NA	1	NA
Bacteria	Mycobacteriaceae	7	3	1	6	3
Bacteria	Mycoplasmataceae	NA	NA	1	NA	NA
Bacteria	Pasteurellaceae	1	NA	NA	1	NA
Bacteria	Pseudomonadaceae	NA	1	NA	NA	NA
Bacteria	Rickettsiaceae	1	NA	NA	1	NA
Bacteria	Staphylococcaceae	NA	2	NA	NA	NA
Bacteria	Yersiniaceae	4	NA	NA	4	3
Helminth	Ancylostomatidae	NA	3	NA	1	4
Helminth	Angiostrongylidae	NA	1	NA	NA	1
Helminth	Anoplocephalidae	2	1	1	NA	NA
Helminth	Ascarididae	NA	5	NA	1	1
Helminth	Brachylaimidae	NA	2	NA	NA	1
Helminth	Capillariidae	NA	5	NA	2	5
Helminth	Centrorhynchidae	NA	1	NA	NA	NA
Helminth	Chabertiidae	1	NA	3	1	NA
Helminth	Crenosomatidae	NA	1	NA	NA	1
Helminth	Cyathocotylidae	NA	1	NA	NA	NA
Helminth	Dicrocoeliidae	1	3	NA	1	1
Helminth	Dictyocaulidae	3	NA	1	NA	NA
Helminth	Dilepididae	NA	NA	NA	NA	1
Helminth	Dioctophymatidae	NA	1	NA	NA	NA
Helminth	Diphyllobothriidae	NA	1	NA	1	1
Helminth	Diplostomatidae	NA	5	NA	NA	NA
Helminth	Diplostomidae	NA	1	NA	NA	3
Helminth	Dipylidiidae	NA	NA	NA	NA	4
Helminth	Dracunculidae	NA	1	NA	NA	NA
Helminth	Echinochasmidae	NA	1	NA	NA	1
Helminth	Echinorhynchidae	NA	1	NA	NA	NA
Helminth	Echinostomatidae	NA	2	NA	NA	1
Helminth	Fasciolidae	2	NA	NA	3	NA
Helminth	Filariidae	NA	1	NA	NA	NA

Helminth	Gnathostomatidae	NA	1	NA	1	NA
Helminth	Gongylonematidae	NA	1	NA	1	NA
Helminth	Haemonchidae	15	NA	12	NA	NA
Helminth	Heterophyidae	NA	10	NA	NA	4
Helminth	Hymenolepididae	NA	NA	NA	NA	1
Helminth	Kathlaniidae	NA	1	NA	NA	NA
Helminth	Lecithodendriidae	NA	1	NA	NA	NA
Helminth	Mesocestoididae	NA	2	NA	NA	2
Helminth	Metastrongylidae	NA	1	NA	6	NA
Helminth	Microphallidae	NA	3	NA	NA	NA
Helminth	Molineidae	1	2	9	NA	4
Helminth	Oligacanthorhynchidae	NA	1	NA	1	3
Helminth	Onchocercidae	4	5	NA	1	4
Helminth	Opisthorchiidae	NA	2	NA	NA	5
Helminth	Oxyuridae	NA	NA	3	NA	NA
Helminth	Paramphistomidae	1	NA	NA	NA	NA
Helminth	Physalopteridae	NA	2	NA	NA	2
Helminth	Plagiorchiidae	NA	1	NA	NA	1
Helminth	Plagiorhynchidae	NA	NA	NA	NA	1
Helminth	Protostrongylidae	3	NA	4	NA	NA
Helminth	Psilostomatidae	NA	1	NA	NA	NA
Helminth	Rictulariidae	NA	NA	NA	NA	1
Helminth	Schistosomatidae	NA	1	NA	NA	NA
Helminth	Spirocercidae	NA	NA	NA	2	NA
Helminth	Strongyloididae	NA	NA	NA	NA	1
Helminth	Subuluridae	NA	NA	NA	NA	1
Helminth	Taeniidae	4	1	NA	3	9
Helminth	Thelaziidae	NA	NA	NA	NA	2
Helminth	Toxocaridae	NA	1	NA	NA	2
Helminth	Trichinellidae	NA	2	NA	3	4
Helminth	Trichostrongylidae	1	NA	5	NA	NA
Helminth	Trichuridae	1	NA	3	1	1
Helminth	Troglotrematidae	NA	3	NA	NA	1
Protozoa	Babesiidae	2	1	2	NA	1

Protozoa	Cryptosporidiidae	1	1	NA	NA	1
Protozoa	Eimeriidae	NA	2	NA	NA	1
Protozoa	Hepatozoidae	NA	1	NA	NA	1
Protozoa	Hexamitidae	1	NA	NA	NA	NA
Protozoa	Sarcocystidae	5	4	3	1	4
Protozoa	Theileriidae	1	NA	NA	NA	NA
Protozoa	Trypanosomatidae	NA	1	NA	NA	1
Virus	Adenoviridae	NA	1	NA	NA	1
Virus	Caliciviridae	NA	NA	NA	NA	1
Virus	Coronaviridae	NA	1	NA	NA	NA
Virus	Flaviviridae	3	2	NA	3	NA
Virus	Herpesviridae	8	1	6	1	1
Virus	Nairoviridae	1	NA	NA	1	NA
Virus	Orthomyxoviridae	NA	1	NA	1	NA
Virus	Paramyxoviridae	1	1	1	NA	1
Virus	Parvoviridae	NA	1	NA	1	1
Virus	Peribunyaviridae	2	NA	NA	2	NA
Virus	Phenuiviridae	1	NA	NA	1	NA
Virus	Pneumoviridae	1	NA	1	NA	NA
Virus	Retroviridae	1	NA	1	NA	NA
Virus	Rhabdoviridae	2	1	NA	2	1
Virus	Sedoreoviridae	1	NA	NA	NA	NA
Virus	Togaviridae	1	NA	NA	1	NA

Table A3. Coefficients and *p*-values (rounded to three decimal places) from linear regressions used in enemy release analyses. PSR = parasite species richness. Range area log transformed. See Methods for definitions of predictors and responses.

**Predictor**	**Response**			
	Proportional change in PSR	Beta diversity	Turnover	Nestedness
Time since invasion	5.21×10^{-5} (p=0.870)	-5.92×10^{-5} (p=0.853)	-3.08×10^{-4} (p=0.118)	3.08×10^{-4} (p=0.118)
Population density	0.045 (p=0.341)	0.060 (p=0.249)	0.048 (p=0.342)	-0.048 (p=0.342)
Range area	-0.042 (p=0.196)	-0.047 (p=0.121)	-0.030 (p=0.240)	0.030 (p=0.240)
Environmental dissimilarity	0.736 (p=0.359)	0.536 (p=0.524)	-0.343 (p=0.601)	0.343 (p=0.601)

Table A4. Quantile values for z-scores from **(a)** random acquisition null model and **(b)** random-relative-to-associations (RRA) null model, using minimum pairwise distance (PD). NAs indicate no loss and/or acquisition of a parasite type by the focal host species, meaning null model z-scores (and thus their quantile values) could not be calculated.

(a) Random acquisition null model

	Cervus elaphus	*Procyon lotor*	*Rupicapra rupicapra*	*Sus scrofa*	*Vulpes vulpes*
Arthropod	0.003	0.001	NA	0.004	0.003
Bacteria	0.050	0.082	0.057	NA	0.006
Helminth	0.012	0.001	NA	0.012	0.003
Protozoa	0.352	NA	0.837	NA	NA
Virus	0.119	0.001	0.044	0.084	NA

(b) Random-relative-to-associations null model

	Cervus elaphus	*Procyon lotor*	*Rupicapra rupicapra*	*Sus scrofa*	*Vulpes vulpes*
Arthropod	0.01	0.01	NA	0.01	0.01
Bacteria	0.08	0.11	0.01	NA	0.01
Helminth	0.08	0.01	NA	0.02	0.01
Protozoa	0.26	NA	0.28	NA	NA
Virus	0.25	0.01	0.16	0.10	NA
